# Genome-Wide Characterization of B-Box Gene Family and Its Roles in Responses to Light Quality and Cold Stress in Tomato

**DOI:** 10.3389/fpls.2021.698525

**Published:** 2021-07-05

**Authors:** Xin Bu, Xiujie Wang, Jiarong Yan, Ying Zhang, Shunyuan Zhou, Xin Sun, Youxin Yang, Golam Jalal Ahammed, Yufeng Liu, Mingfang Qi, Feng Wang, Tianlai Li

**Affiliations:** ^1^College of Horticulture, Shenyang Agricultural University, Shenyang, China; ^2^Key Laboratory of Protected Horticulture, Ministry of Education, Shenyang, China; ^3^National & Local Joint Engineering Research Center of Northern Horticultural Facilities Design & Application Technology, Shenyang, China; ^4^College of Land and Environment, Shenyang Agricultural University, Shenyang, China; ^5^College of Agronomy, Jiangxi Agricultural University, Nanchang, China; ^6^College of Forestry, Henan University of Science and Technology, Luoyang, China

**Keywords:** BBX, light, cold stress, *Solanum lycopersicum*, photoinhibition

## Abstract

Perceiving incoming environmental information is critical for optimizing plant growth and development. Multiple B-box proteins (BBXs) play essential roles in light-dependent developmental processes in plants. However, whether BBXs function as a signal integrator between light and temperature in tomato plants remains elusive. In this study, 31 *SlBBX* genes were identified from the newly released tomato (*Solanum lycopersicum*) genome sequences and were clustered into five subgroups. Gene structure and protein motif analyses showed relatively high conservation of closely clustered *SlBBX* genes within each subgroup; however, genome mapping analysis indicated the uneven distribution of the *SlBBX* genes on tomato chromosomes. Promoter *cis*-regulatory elements prediction and gene expression indicated that *SlBBX* genes were highly responsive to light, hormones, and stress conditions. Reverse genetic approaches revealed that disruption of *SlBBX7, SlBBX9*, and *SlBBX20* largely suppressed the cold tolerance of tomato plants. Furthermore, the impairment of *SlBBX7, SlBBX9*, and *SlBBX20* suppressed the photosynthetic response immediately after cold stress. Due to the impairment of non-photochemical quenching (NPQ), the excess photon energy and electron flow excited by low temperature were not consumed in *SlBBX7-, SlBBX9-*, and *SlBBX20-* silenced plants, leading to the over reduction of electron carriers and damage of the photosystem. Our study emphasized the positive roles of light signaling transcription factors SlBBXs in cold tolerance in tomato plants, which may improve the current understanding of how plants integrate light and temperature signals to adapt to adverse environments.

## Highlight

- SlBBXs function as a signal integrator between light and temperature in tomato.

## Introduction

The B-box (BBX) proteins represent a unique class of zinc-finger transcription factors (TFs) that possess single or double B-box domains in their N termini and a CCT (CO, CO-like, and TOC1) domain in their C termini in some cases (Gangappa and Botto, 2014). The B-box domains are of two classes, and each of them coordinates two zinc atoms (Khanna et al., 2009). The dissimilarities in the consensus sequences of the two B-box domains are the results of evolution through the segmental duplication and deletion events (Crocco and Botto, 2013). Studies suggest that the highly conserved CCT domain is important for transcriptional regulation and nuclear transport (Gendron et al., 2012). Furthermore, the valine-proline (VP) motif of six amino acids (G-I/V-V-P-S/T-F) contained by some BBX proteins, plays a crucial role in the interaction with CONSTITUTIVELY PHOTOMORPHOGENIC 1 (COP1) (Holm et al., 2001; Datta et al., 2006). Based on the domain structures, 32 BBX proteins are divided into five subfamilies in Arabidopsis (Crocco and Botto, 2013; Gangappa and Botto, 2014).

A variety of wavelength-specific photoreceptors are involved in perceiving the light signals in plants, including phytochromes (phys), cryptochromes (CRYs), phototropins (PHOTs), ZEITLUPE family members, and UV-B resistance locus 8 (UVR8) (Galvao and Fankhauser, 2015; Paik and Huq, 2019). Light-activated photoreceptors inhibit the COP1-SUPPRESSOR OF PHYA-105 (SPA) E3 ubiquitin ligase complex, which functions for the degradation of the positive regulators of photomorphogenesis (Galvao and Fankhauser, 2015; Paik and Huq, 2019). Notably, HY5, a target of COP1-mediated protein degradation, plays a vital role in light-regulated plant growth and development (Osterlund et al., 2000; Ahammed et al., 2020). Thus, the light-dependent regulation of COP1–HY5 mediates the plant developmental transition from dark to light.

Upon light irradiation, BBX21 directly binds to *BBX22, HY5* and its own promoter regions and activates its transcription (Xu et al., 2016, 2018; Xu, 2020). Moreover, both BBX21 and HY5 can associate with the *BBX11* promoter to promote its transcription, while BBX11 binds to the *HY5* promoter to activate its transcription (Zhao et al., 2020). Thus, these three TFs (BBX21, HY5, and BBX11) form a positive feedback loop that precisely regulates plant photomorphogenesis. OsBBX14 induces *OsHY5L1* gene expression to stimulate photomorphogenesis in rice (Bai et al., 2019). MdBBX37 associates with *MdHY5* promoter to inhibit its expression in apple (An et al., 2019a). Additionally, MdBBX22 and MdBBX25/MdCOL4 bind to the MdHY5 promoter to increase and decrease the transcriptional activation of MdHY5, respectively (An et al., 2019b). Both PpBBX16 and PpBBX18 interact with PpHY5 to increase the biochemical activity of PpHY5, while PpHY5 binds to the promoter region of *PpBBX18* to promote the transcription of *PpBBX18* in pear (Bai et al., 2019a,b). Furthermore, the interaction of PpBBX21 with PpHY5 and PpBBX18 affects the bioactive heterodimer formation of PpHY5-PpBBX18 (Bai et al., 2019b). Tomato RIPENING INHIBITOR (SlRIN) binds to the ripening-induced *SlBBXs* (i.e., *SlBBX19, SlBBX20*, and *SlBBX26*) promoter (Lira et al., 2020). SlBBX20 modulates carotenoid biosynthesis by directly activating *PHYTOENE SYNTHASE 1*, and is targeted for 26S proteasome-mediated degradation in tomato (Xiong et al., 2019). Therefore, specific BBXs and HY5 constitute an important regulatory network to precisely control normal plant growth and development.

In the darkness, CO/BBX1, BBX4, BBX10, BBX19, BBX20, BBX21, BBX22, BBX23, BBX24, BBX25, BBX28, and BBX29 are ubiquitinated by COP1 and subsequently degraded by the 26S proteasome system (Fan et al., 2012; Gangappa et al., 2013; Wang et al., 2015; Xu et al., 2016; Zhang et al., 2017; Lin et al., 2018; Ordoñez-Herrera et al., 2018; Heng et al., 2019a; Song et al., 2020). Moreover, BBX2 to 9 and BBX13 to 16 interacts with COP1 *in vitro*, indicating a role for COP1 in controlling the stability of these proteins in darkness (Ordoñez-Herrera et al., 2018). Nevertheless, COP1 preferentially stabilizes BBX11 instead of promoting its degradation (Zhao et al., 2020), which suggests that COP1 likely regulates a yet unidentified protein degrading BBX11. These studies suggest that numerous BBX proteins, along with COP1 and HY5, play critical roles in light-dependent development in plants.

BBX proteins also play vital roles in regulatory networks that control plant adaption to abiotic stress. Previous studies show that both BBX5 and BBX21 positively regulate plant tolerance to drought and salt stress in Arabidopsis (Nagaoka and Takano, 2003; Min et al., 2015). BBX24/STO directly interacts with H-protein promoter binding factor 1 (HPPBF-1), which is a salt-responsive MYB transcription factor, to enhance the root growth and salt tolerance in Arabidopsis (Nagaoka and Takano, 2003). CmBBX22 also positively regulates the plant drought tolerance (Liu Y. N. et al., 2019). In addition, MdBBX10 enhances tolerance to salt and drought by modulating ABA signaling and ROS accumulation (Liu X. et al., 2019). In Arabidopsis, BBX18 and BBX23 control thermomorphogenesis (Ding et al., 2018). Both *MdBBX20* and *MaCOL1* are responsive to low temperature in apple and banana, respectively (Chen et al., 2012; Fang et al., 2019). Furthermore, *ZFPL*, a homologous gene of *AtBBX32*, enhances cold tolerance in the grapevine (Takuhara et al., 2011). *CmBBX24* also increases plant cold tolerance in *Chrysanthemum* (Yang et al., 2014). However, whether SlBBXs are involved in light and cold response in tomato remains to be explored.

In the present study, 31 *SlBBX* genes were identified and characterized in tomato. Gene distribution, synteny analyses, the architecture of exon-intron and motifs differences were investigated. Promoter and gene expression analysis showed that *SlBBXs* played important roles in plant response to light and low temperature signaling. Meanwhile, we found that the impairment of *SlBBX7, SlBBX9*, and *SlBBX20* suppressed the photosynthetic response and non-photochemical quenching (NPQ) immediately after cold stress, which resulted in excess photon energy and electron flow in *SlBBX7-, SlBBX9-*, and *SlBBX20-* silenced plants, leading to the overreduction of electron carriers and damage of photosystem. Our study indicated that light signaling transcription factors *SlBBX7-, SlBBX9-*, and *SlBBX20-* play positive roles in cold tolerance in tomato plants, which may improve the current understanding of plant integrated light and temperature signals to adapt the adverse environments.

## Materials and Methods

### Genome-Wide Identification of *SlBBX* Genes in Tomato

The BBXs proteins in tomato were identified based on protein homology searches from Arabidopsis using the Hidden Markov Model (HMM) as previously described (Upadhyay and Mattoo, 2018). The protein sequence of Arabidopsis *BBXs* were downloaded from the TAIR database (https://www.arabidopsis.org/). Tomato BBX proteins were searched and downloaded from three public databases, including the NCBI database (http://www.ncbi.nlm.nih.gov/), the Phytozome 13.0 database (https://phytozome.jgi.doe.gov/pz/portal.html), and the Sol Genomics Network tomato database (version ITAG 4.0, https://solgenomics.net/). Tomato BBX proteins resulting from both searches (E-value, 10^−5^) were pooled and redundant sequences were removed. InterProScan database (http://www.ebi.ac.uk/interpro/), SMART (http://smart.embl-heidelberg.de/), and Conserved Domains Database (http://www.ncbi.nlm.nih.gov/cdd/) were used to further confirm the existence of B-box domain in retrieved BBX sequences.

### Protein Properties, Multiple Sequence Alignment, Phylogenetic, and Conserved Motifs Analysis

The various physiochemical properties of tomato BBX proteins, such as MW, polypeptide length, pI, instability index, aliphatic index, and GRAVY were investigated using the ExPASy online tool (http://web.expasy.org/protparam/). To estimate the subcellular localization of tomato BBX proteins, we used CELLO v.2.5: sub-cellular localization predictor (http://cello.life.nctu.edu.tw/) (Yu et al., 2006) and pSORT prediction software (http://www.genscript.com/wolf-psort.html) (Horton et al., 2007). Open Reading Frame (ORF) numbers were calculated using the NCBI website (https://www.ncbi.nlm.nih.gov/orffinder/).

A multiple sequence alignment of the identified tomato BBX proteins and the known BBX families from Arabidopsis, rice, and potato, was performed with the MUSCLE (https://www.ebi.ac.uk/Tools/msa/muscle/) (Edgar, 2004) and DNAMAN software (Version 5.2.2). We constructed phylogenetic tree using MEGA 7.0 with 1,000 bootstrap value and the maximum likelihood method (Kumar et al., 2016), and the phylogenetic tree was displayed with an online website Evolview (http://www.evolgenius.info/evolview/#mytrees/) (Zhang et al., 2012).

The presence of conserved BBX_N and CCT_C—domains were identified by NCBI (https://www.ncbi.nlm.nih.gov/Structure/cdd/wrpsb.cgi), and drawn with IBS software (Illustrator for Biological Sequences, http://ibs.biocuckoo.org/online.php) (Ren et al., 2009). We performed the protein structural motif annotation using the Meme program (http://meme-suite.org/tools/meme) (Bailey et al., 2009; Upadhyay and Mattoo, 2018) and limited our search to a maximum of 20 motifs.

### Chromosomal Location, Gene Structure, and Synteny Analysis

*SlBBX* genes were mapped to tomato chromosomes according to the Phytozome 13.0 database with the MapChart software. The chromosome distribution diagram was drawn by the online website MG2C (http://mg2c.iask.in/mg2c_v2.1/) with the information from Sol Genomics Network (http://www.solgenomics.net).

Genomic DNA sequence and CDS corresponding to each identified SlBBX gene were retrieved from the tomato genome database. Intron-exon were displayed by comparing CDS to genomic sequences with the gene structure display server (GSDS, http://gsds.cbi.pku.edu.cn/) (Hu et al., 2015).

The syntenic blocks were designed from the Plant Genome Duplication Database (http://chibba.agtec.uga.edu/duplication/). The synteny figures were drawn by Circos-0.69 (http://circos.ca/) with E-value setting to 1e^−10^ and output format as tabular (-m 8).

### *Cis*-Elements of Promoters Analysis

To identify potential light-, stress-, hormone-, and development-related *cis*-elements, the 2,000-bp genomic DNA sequence upstream of the start codon (ATG) of *SlBBX* genes were obtained from the tomato genome database. The *cis*-elements in these *SlBBX* genes promoter were identified by using the Plant Cis-Acting Regulatory Element (PlantCARE; http://bioinformatics.psb.ugent.be/webtools/plantcare/html/) (Lescot et al., 2002).

### Plant Material and Growth Conditions

Seeds of tomato (*Solanum lycopersicum*) in the cv “Ailsa Craig” (Accession: LA2838A) background were obtained from the Tomato Genetics Resource Center (http://tgrc.ucdavis.edu) as previously reported (Wang et al., 2016). Seedlings, which grown in pots with a mixture of one part vermiculite to three parts peat, receive Hoagland nutrient solution. The growth conditions for tomato seedlings were 25/20°C (day/night) temperature with a 12 h photoperiod, light intensity of 600 μmol m^−2^ s^−1^, and 65% relative humidity. Tobacco rattle virus-based vectors (pTRV1/2) were used for the VIGS of *SlBBX* genes in tomato with the specific PCR-amplified primers listed in Supplementary Table 3. The PCR-amplified fragment was cloned into the pTRV2 vector. The empty vector of pTRV*2* was used as the control. All constructs were confirmed by sequencing and subsequently transformed into *Agrobacterium tumefaciens* strain GV3101. VIGS was performed as described previously (Wang et al., 2016, 2019). The inoculated plants were grown under a 12 h photoperiod at 22/20°C (day/night).

### Light and Cold Treatments

The six-leaf stage plants were used for all experiments. Plants grown under white light were exposed to a temperature of 25 or 4°C for the control or cold treatment, respectively, in environment-controlled growth chambers (Ningbo Jiangnan instrument factory, Ningbo, China). For different light quality treatments, plants were exposed to dark (D), white light (W), or different wavelength [purple (P), 394 nm; blue (B), 450 nm; green (G), 522 nm; yellow (Y), 594 nm; red (R), 660 nm and far-red (FR), 735 nm, Philips] light from 6:00 a.m. to 6:00 p.m. The light intensity was 100 μmol m^−2^ s^−1^. The Lighting Passport (Asensetek, Model No. ALP-01, Taiwan) was used to measure light intensity and light quality as a previous study (Wang et al., 2020b).

### Gene Expression Analysis

Total RNA was extracted from tomato leaves as described previously (Wang et al., 2020a). The cDNA template for real-time qRT-PCR was synthesized using a Rever-Tra Ace qPCR RT Kit with a genomic DNA-removing enzyme (Toyobo). qRT-PCR experiments were carried out with an SYBR Green PCR Master Mix Kit (TaKaRa) using an Applied Biosystems 7500 Real-Time PCR System (qTOWER^3^G, Germany). The primer sequences are listed in Supplementary Table 2. The PCR was run at 95°C for 3 min, followed by 40 cycles of 30 s at 95^o^C, 30 s at 58°C, and 1 min at 72°C. The tomato *ACTIN* gene was used as an internal control. The relative gene expression was calculated as described previously (Livak and Schmittgen, 2001).

### Cold Tolerance Assays

The relative electrolyte leakage (REL), an indicator for cellular membrane permeability, was measured as described previously (Cao et al., 2007). Chlorophyll fluorescence and P700 absorption changes were determined simultaneously using a DUAL-PAM-100 (Heinz Walz, Effeltrich, Germany) as previously described (Wang et al., 2020b). Before measurements, plants were dark-acclimated for 30 min. PSII and PSI parameters were calculated as follows: the maximum quantum yield of PSII (Fv/Fm) as (Fm - Fo)/Fm, the effective quantum yield of PSII [Y(II)] as (Fm' - Fs)/Fm', the quantum yield of non-regulated energy dissipation of PSII [Y(NO)] as Fs/Fm, the quantum yield of regulated energy dissipation of PSII [Y(NPQ)] as 1- Y(II) - Y(NO), NPQ as (Fm – Fm')/Fm', photochemical quenching coefficient (qP) as (Fm' - Fs)/(Fm' – Fo'), the donor limitation of PSI [Y(ND)] as 1 – P700red, or P/Pm, the acceptor side limitation of PSI [Y(NA)] as (Pm – Pm')/Pm, the quantum yield of PSI [Y(I)] as 1 – Y(ND) – Y(NA), where Fm and Fo represent the maximum and minimum fluoresce yields, whereas Pm and Pm′ represent the P700 signals recorded just before (P) then briefly after the onset of a saturating pulse (Pm′). P700red, which represents the fraction of overall P700 that is reduced in a given state, was determined with the help of a saturation pulse. The electron transport rate (ETRI or ETRII) was calculated as 0.5 × abs I × Y(I) or 0.5 × abs I × Y(II), where 0.5 is the proportion of absorbed light reaching PSI or PSII, and abs I is absorbed irradiance taken as 0.84 of incident irradiance (Wang et al., 2020b).

### Statistical Analysis

All statistics were calculated using SPSS software. To determine statistical significance, we employed Tukey's test. A value of *P* < 0.05 was considered to indicate statistical significance.

## Results

### Identification and Characterization of *SlBBX* Genes in Tomato

Based on the gene annotation as well as the conserved B-box motif characteristic of the BBX members, a total of 31 *SlBBX* genes were identified. The detailed information of each *SlBBX* is presented in Table 1. The lengths of amino acids (AA) of 31 SlBBXs range from 88 aa (*SlBBX18*) to 475 aa (*SlBBX27*). Thus, varied molecular weight (MW) and theoretical isoelectric point (pI) were observed among SlBBX proteins. The MW of SlBBXs varies from 9.57 (SlBBX18) to 53.14 kDa (SlBBX27). The pI ranged from 4.25 (SlBBX5 and SlBBX7) to 9.28 (SlBBX26), with 74.2% SlBBXs with a pI lower than seven, which indicated that most of the SlBBX proteins were acidic in nature. The pI ranged from 4 to 9 in SlBBX proteins contained one (single) or two (double) B-box domains; however, the pI ranged from 4 to 7 when SlBBX proteins contained a CCT domain (Supplementary Figure 1), suggesting that the CCT domain in SlBBX proteins may decrease its pI. The majority of SlBBXs were grouped into unstable proteins because their instability index was greater than 40, except for *SlBBX6* in this family (Table 1). The predicted aliphatic index ranged from 50.05 to 97.43 in SlBBX proteins. All SlBBX proteins, with the exception of SlBBX18, were predicted to be hydrophilic due to the GRAVY value (<0). Subcellular localization predicted that 23 SlBBX proteins are located in nuclei. Among the rest 8 tomato BBXs, five (SlBBX5, SlBBX6, SlBBX17, SlBBX25, and SlBBX31), two (SlBBX16 and SlBBX18), and one (SlBBX19) SlBBX proteins are located in chloroplast, cytoplasm, and peroxisome, respectively (Table 1).

**Table 1:** Nomenclature, identification, chromosomal location, theoretical isoelectric point (pI), molecular weight (MW), CDS, peptide length, number of exon and intron, instability and aliphatic index, gravy and subcellular localization of BBX gene family in tomato.

**Name**	**Gene locus ID**	**pI**	**MW(kDa)**	**AA**	**Exon**	**intron**	**Instability index**	**Aliphatic index**	**Gravy**	**Subcellular Localization**
SlBBX1	Solyc02g089520	4.89	45.69	409	3	2	41.8	66.06	−0.630	nucl: 14
SlBBX2	Solyc02g089500	8.16	15.18	142	2	1	72.59	70.92	−0.015	nucl: 6, mito: 5, chlo: 1, cyto: 1, extr: 1
SlBBX3	Solyc02g089540	5.76	43.44	391	3	2	41.06	64.14	−0.625	nucl: 10, chlo: 2, cyto: 1, cysk: 1
SlBBX4	Solyc08g006530	5.15	38.66	349	3	2	51.34	63.75	−0.602	nucl: 8, chlo: 3, cyto: 3
SlBBX5	Solyc12g096500	4.25	29.71	358	3	2	48.52	62.96	−0.503	chlo: 10, nucl: 3, cyto: 1
SlBBX6	Solyc07g006630	6.82	42.61	386	2	1	38.63	71.04	−0.364	chlo: 7, cyto: 4, nucl: 1, mito: 1, extr: 1
SlBBX7	Solyc12g006240	4.25	29.71	269	3	2	49.44	66.90	−0.604	nucl: 8, extr: 3, chlo: 2, cyto: 1
SlBBX8	Solyc05g020020	5.49	44.53	410	5	4	58.86	63.54	−0.482	nucl: 12, cyto: 2
SlBBX9	Solyc07g045180	5.32	46.14	418	5	4	55.69	57.85	−0.524	nucl: 14
SlBBX10	Solyc05g046040	4.73	46.27	416	4	3	53.21	65.66	−0.551	nucl: 12, cyto: 1, cysk: 1
SlBBX11	Solyc09g074560	6.16	42.23	373	4	3	51.28	61.93	−0.765	nucl: 10, cyto: 2, chlo: 1, vacu: 1
SlBBX12	Solyc05g024010	7.17	49.71	452	4	3	49.44	66.90	−0.604	nucl: 11, cyto: 1, extr: 1, vacu: 1
SlBBX13	Solyc04g007210	5.13	48.72	428	2	1	45.21	61.71	−0.774	nucl: 11, chlo: 1, mito: 1, cysk: 1
SlBBX14	Solyc03g119540	4.92	46.70	408	2	1	49.49	65.78	−0.767	nucl: 10, chlo: 3, mito: 1
SlBBX15	Solyc05g009310	5.54	49.82	437	2	1	40.72	68.47	−0.774	nucl: 6, mito: 3, chlo: 2, cyto: 2, cysk: 1
SlBBX16	Solyc12g005750	7.85	12.72	109	1	0	42.52	97.43	−0.037	cyto: 8, nucl: 3, mito: 1, cysk: 1, golg: 1
SlBBX17	Solyc07g052620	8.26	14.52	130	1	0	53.75	70.62	−0.370	chlo: 7, nucl: 3, cyto: 2, plas: 1, extr: 1
SlBBX18	Solyc02g084420	5.57	9.57	96	4	3	45.42	78.75	0.194	cyto: 11, chlo: 1, mito: 1, extr: 1
SlBBX19	Solyc01g110370	5.17	26.88	241	6	5	54.64	76.89	−0.447	pero: 9, cyto: 2.5, cyto_nucl: 2, chlo: 1, golg: 1
SlBBX20	Solyc12g089240	7.40	36.4	329	3	2	54.04	67.54	−0.491	nucl: 10, cyto: 2, chlo: 1, extr: 1
SlBBX21	Solyc04g081020	7.61	33.27	299	3	2	62.74	70.43	−0.472	nucl: 6, cyto: 4, extr: 2, chlo: 1, cysk: 1
SlBBX22	Solyc07g062160	4.61	32.07	298	3	2	57.90	68.09	−0.360	nucl: 12, cyto: 1, cysk: 1
SlBBX23	Solyc12g005420	6.23	30.64	282	3	2	54.04	69.22	−0.326	nucl: 10, chlo: 3, extr: 1
SlBBX24	Solyc06g073180	4.74	25.92	233	4	3	54.31	78.76	−0.391	nucl: 7, cyto: 2, cysk: 2, chlo: 1, plas: 1, extr: 1
SlBBX25	Solyc01g110180	5.96	22.62	203	3	2	48.29	68.28	−0.485	nucl: 10, chlo: 1, cyto: 1, extr: 1, vacu: 1
SlBBX26	Solyc10g006750	9.28	12.04	104	2	1	53.39	78.75	−0.180	nucl: 10.5, cyto_nucl: 6.5, extr: 2, cyto: 1.5
SlBBX27	Solyc04g007470	5.94	53.14	475	5	4	58.77	62.84	−0.661	nucl: 14
SlBBX28	Solyc12g005660	4.63	22.27	465	2	1	55.31	69.46	−0.552	chlo: 8, nucl: 2, cyto: 2, extr: 2
SlBBX29	Solyc02g079430	4.49	20.73	185	2	1	68.52	50.05	−1.034	nucl: 8, chlo: 2, mito: 2, cyto: 1, plas: 1
SlBBX30	Solyc06g063280	8.90	28.72	261	1	0	65.08	73.64	−0.268	nucl: 11, cyto: 2, extr: 1
SlBBX31	Solyc07g053140	4.31	28.19	257	2	1	63.17	52.80	−0.840	chlo: 7, nucl: 6, mito: 1

*pI, theoretical isoelectric point; MW, molecular weight; CDS, length of coding sequence; AA, amino acid; gravy, grand average of hydropathicity; nucl, nucleus; mito, mitochondria; chlo, chloroplast; cyto, cytoplasm; extr, extracellular; cysk, cytoskeleton; vacu, vacuole; golg, golgi apparatus; pero, peroxisome; plas, plasma membrane*.

### Protein Sequences and Phylogenetic Analysis of SlBBXs

**Figure 1:**
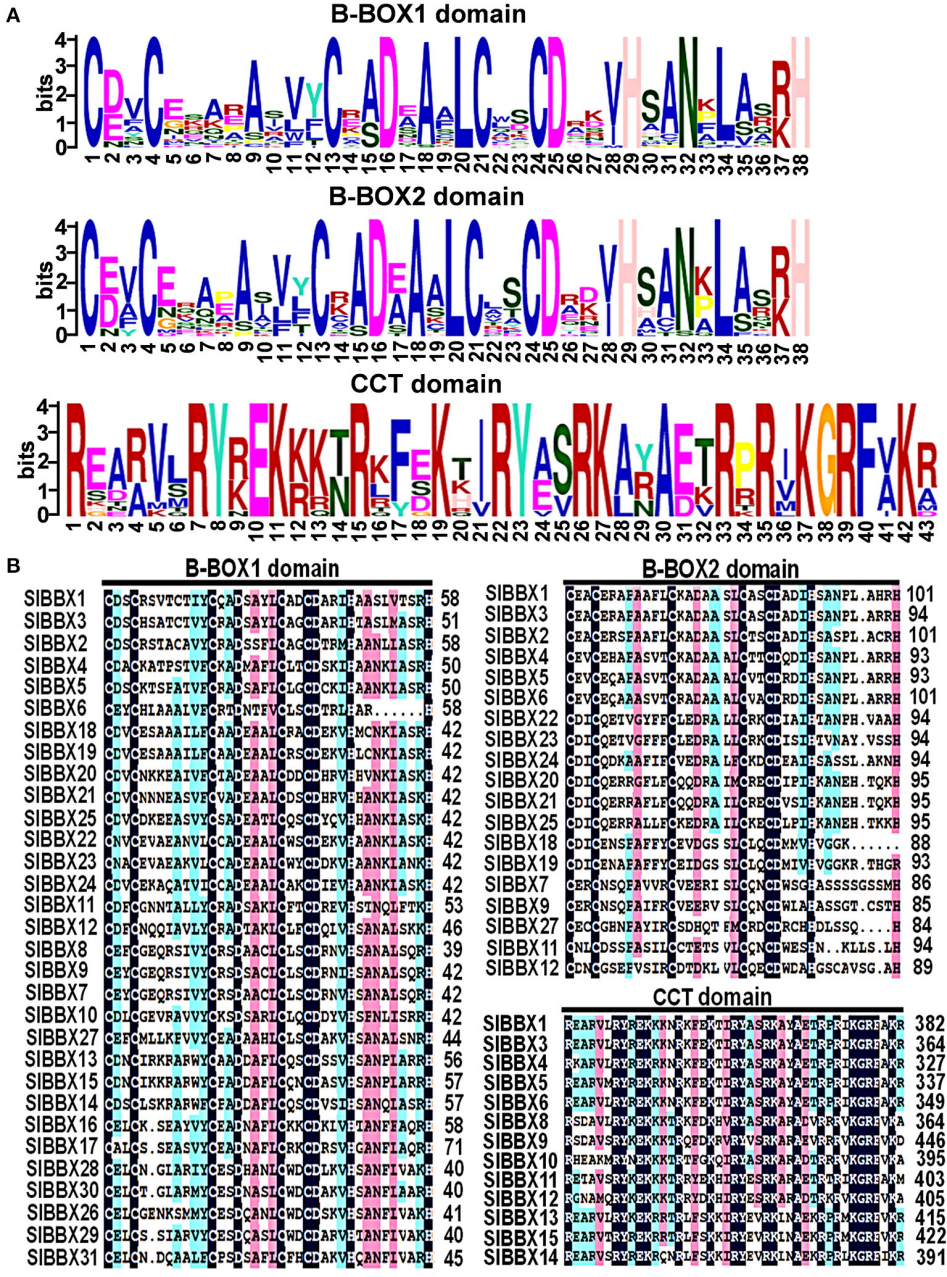
Domain composition of SlBBX proteins. **(A)** The amino acid sequence alignment of the B-box1, B-box2, and CCT domain. The y-axis and x-axis indicated the conservation rate of each amino acid and the conserved sequences of the domain, respectively. **(B)** Multiple sequence alignments of the domains of the SlBBXs. Multiple sequence alignments of the B-box1, B-box2, and CCT domains are shown. The identical conserved amino acids were represented by black and pink shaded.

The domains logos and the sequences of the B-box1, B-box2, and CCT domain of the SlBBX proteins are shown in Figure 1. Eight members out of the 31 SlBBXs, were characterized by the occurrence of two B-box domains and also a conserved CCT domain, whereas four members of them had a valine-proline (VP) motif (Table 2). Only two B-box domains were found in 10 SlBBXs, whereas five members had one B-box domain and also a CCT domain, and eight members had only one B-box domain (Table 2). Among the three domains, we found that each tomato B-box motif contained ~40 residues with the consensus sequence C-X2-C-X8-C-X2-D-X4-C-X2-C-D-X3-H-X8-H (Figure 1). The conserved C, C, D, and H residues ligated two zinc ions (Khanna et al., 2009). Additionally, the consensus sequence of the conserved CCT domain was R-X5-R-Y-X-E-K-X3-R-X3-K-X2-R-Y-X2-R-K-X2-A-X2-R-X-R-X-K-G-R-F-X-K (Figure 1).

**Table 2:** Structure of the tomato BBX proteins.

**Name**	**Gene locus ID**	**AA(aa)**	**Domains**	**B-box1**	**B-box2**	**CCT**	**VP**	**Protein structure**
SlBBX1	Solyc02g089520	409	2B-box+CCT	19–63	59–106	340–382		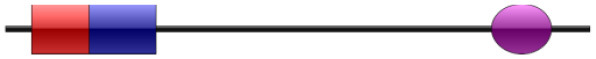
SlBBX2	Solyc02g089500	142	2B-box	19–63	62–106			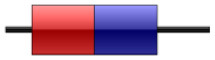
SlBBX3	Solyc02g089540	391	2B-box+CCT	12–56	52–99	322–364	386–391	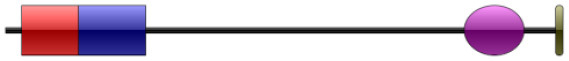
SlBBX4	Solyc08g006530	349	2B-box+CCT	12–55	51–98	285–327	344–349	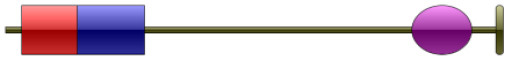
SlBBX5	Solyc12g096500	358	2B-box+CCT	12–55	51–98	295–337	353–358	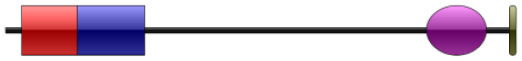
SlBBX6	Solyc07g006630	386	2B-box+CCT	22–63	59–106	307–349	379–384	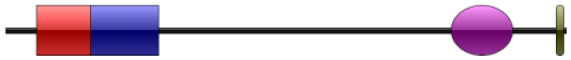
SlBBX7	Solyc12g006240	269	2B-box	4–47	47–73			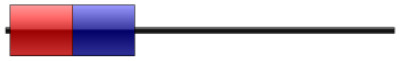
SlBBX8	Solyc05g020020	410	1B-box+CCT	1–44		356–396		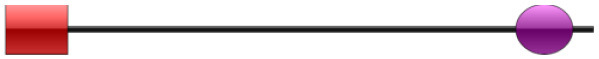
SlBBX9	Solyc07g045180	418	2B-box+CCT	4–47	47–90	361–404		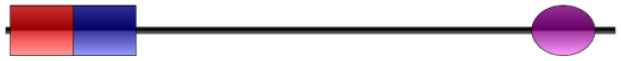
SlBBX10	Solyc05g046040	419	1B-box+CCT	3–47		363–406		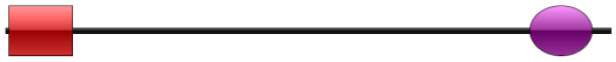
SlBBX11	Solyc09g074560	373	2B-box+CCT	15–58	58–99	322–365		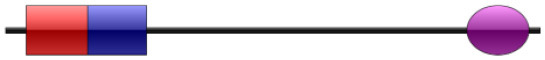
SlBBX12	Solyc05g024010	452	2B-box+CCT	7–39	51–94	404–447		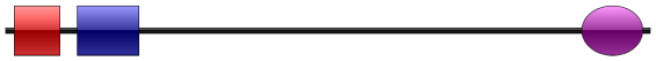
SlBBX13	Solyc04g007210	428	1B-box+CCT	17–61		373–415		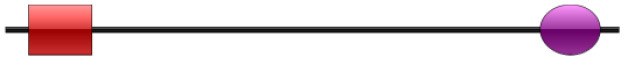
SlBBX14	Solyc03g119540	408	1B-box+CCT	18–62		349–392		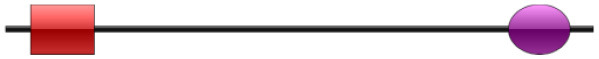
SlBBX15	Solyc05g009310	437	1B-box+CCT	17–61		380–423		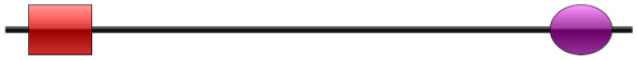
SlBBX16	Solyc12g005750	110	1B-box	21–50				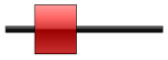
SlBBX17	Solyc07g052620	130	1B-box	35–76				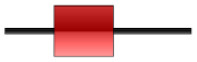
SlBBX18	Solyc02g084420	88	2B-box	2–33	52–84			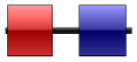
SlBBX19	Solyc01g110370	241	2B-box	2–45	54–96			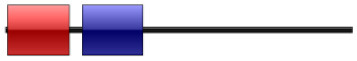
SlBBX20	Solyc12g089240	329	2B-box	5–47	53–100			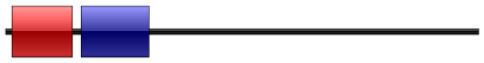
SlBBX21	Solyc04g081020	299	2B-box	5–47	56–100			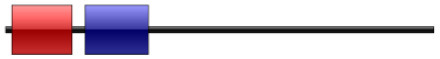
SlBBX22	Solyc07g062160	311	2B-box	5–47	53–99			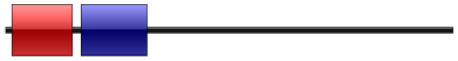
SlBBX23	Solyc12g005420	282	2B-box	5–47	53–99			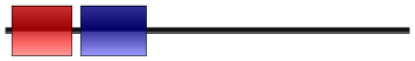
SlBBX24	Solyc06g073180	233	2B-box	5-44	53-98			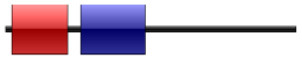
SlBBX25	Solyc01g110180	203	2B-box	3–33	56–100			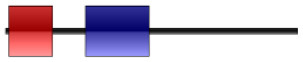
SlBBX26	Solyc10g006750	104	1B-box	4–34				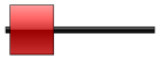
SlBBX27	Solyc04g007470	475	2B-box	7–49	49–87			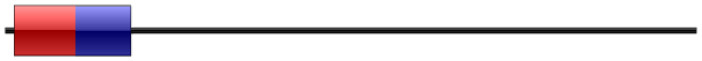
SlBBX28	Solyc12g005660	202	1B-box	4–45				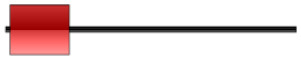
SlBBX29	Solyc02g079430	185	1B-box	1–45				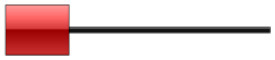
SlBBX30	Solyc06g063280	261	1B-box	4–50				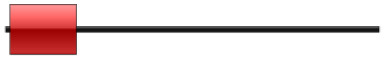
SlBBX31	Solyc07g053140	257	1B-box	4–45				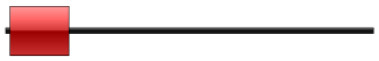

*Numbers indicate the amino acid position of the corresponding conserved domains. The red, blue rectangles, purple circles, and gray rectangles indicate the B-box1, B-box2, CCT domain, and VP domain, respectively*.

**Figure 2:**
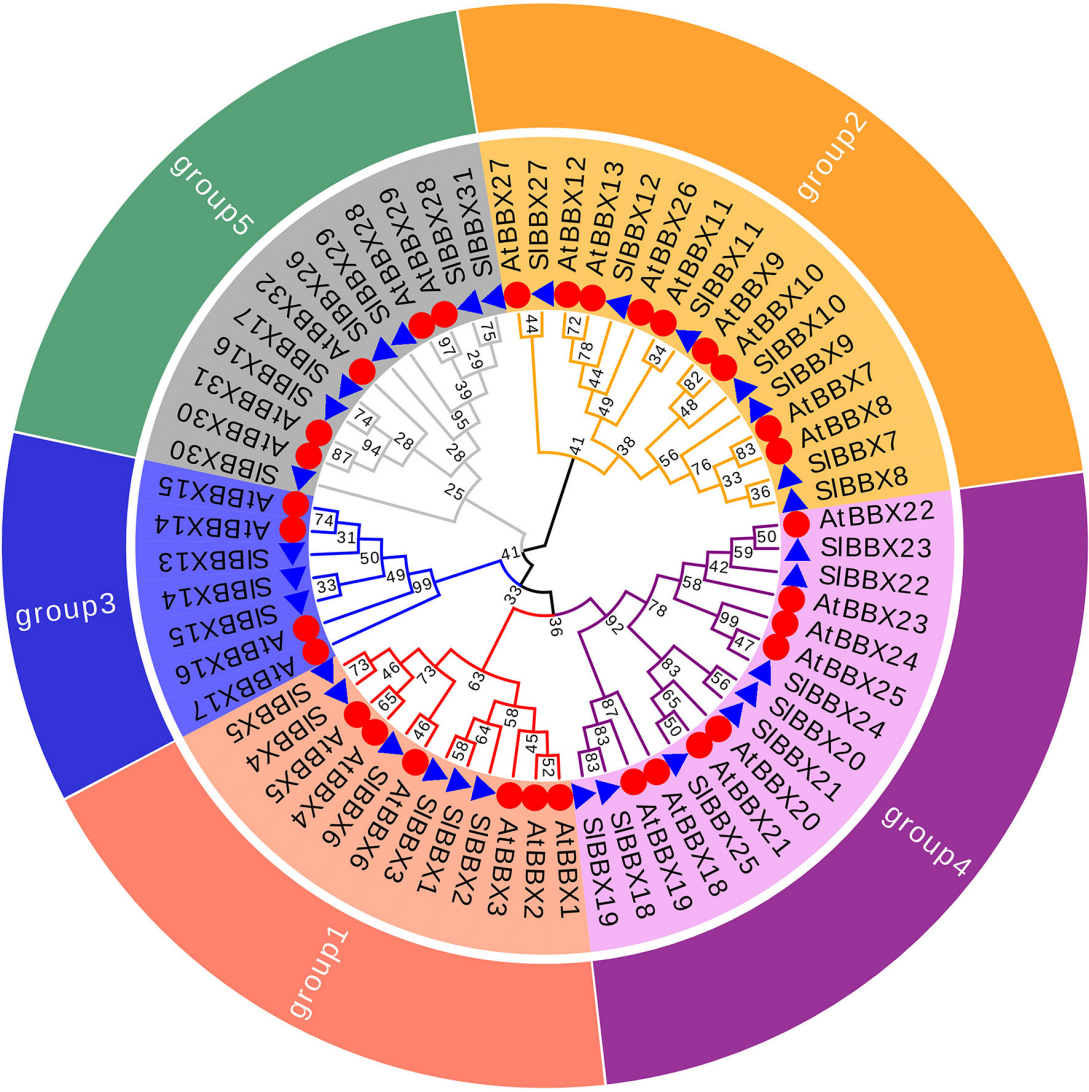
Molecular phylogenetic analysis of *SlBBX* genes in tomato. All SlBBX proteins were divided into five subclasses represented by different colored clusters. Red, orange, bule, purple, and green clusters represent subclasses I, II, III, IV, and V, respectively. The evolutionary history was inferred by using the Maximum Likelihood method using MEGA7 software with 1,000 bootstrap replicates. The red circles and blue triangles represent Arabidopsis and tomato, respectively.

To better reveal the evolutionary relationships, we generated a phylogenetic tree with the known BBX families from Arabidopsis, rice and potato, and the identified tomato BBX protein sequences (Figure 2, Supplementary Figure 2). All sequences of tomato BBX proteins were clustered into five subfamilies (Figure 2). The *BBX* genes in group 1 had two concatenated B-box domains, a CCT domain and a VP motif except for SlBBX1 and SlBBX2, which did not have a VP motif and a CCT domain. The members of group 2 were characterized by two B-box domains and also a CCT domain with the exception for SlBBX7 and SlBBX27, which contained two B-box domains only, and SlBBX8 and SlBBX10, which only had one B-box domain and a CCT domain. In group 3, all the members contained one B-box domain as well as a CCT domain. Group 4 and 5 possessed two and one B-box domain, respectively. Additionally, BBX proteins from two species showed scattered distribution across the branches of the evolutionary tree, which implies that the duplication events occurred after the lineages diverged.

### Gene Structure, Conserved Motifs, Chromosomal Localization, and Synteny Analysis of *SlBBXs*

**Figure 3:**
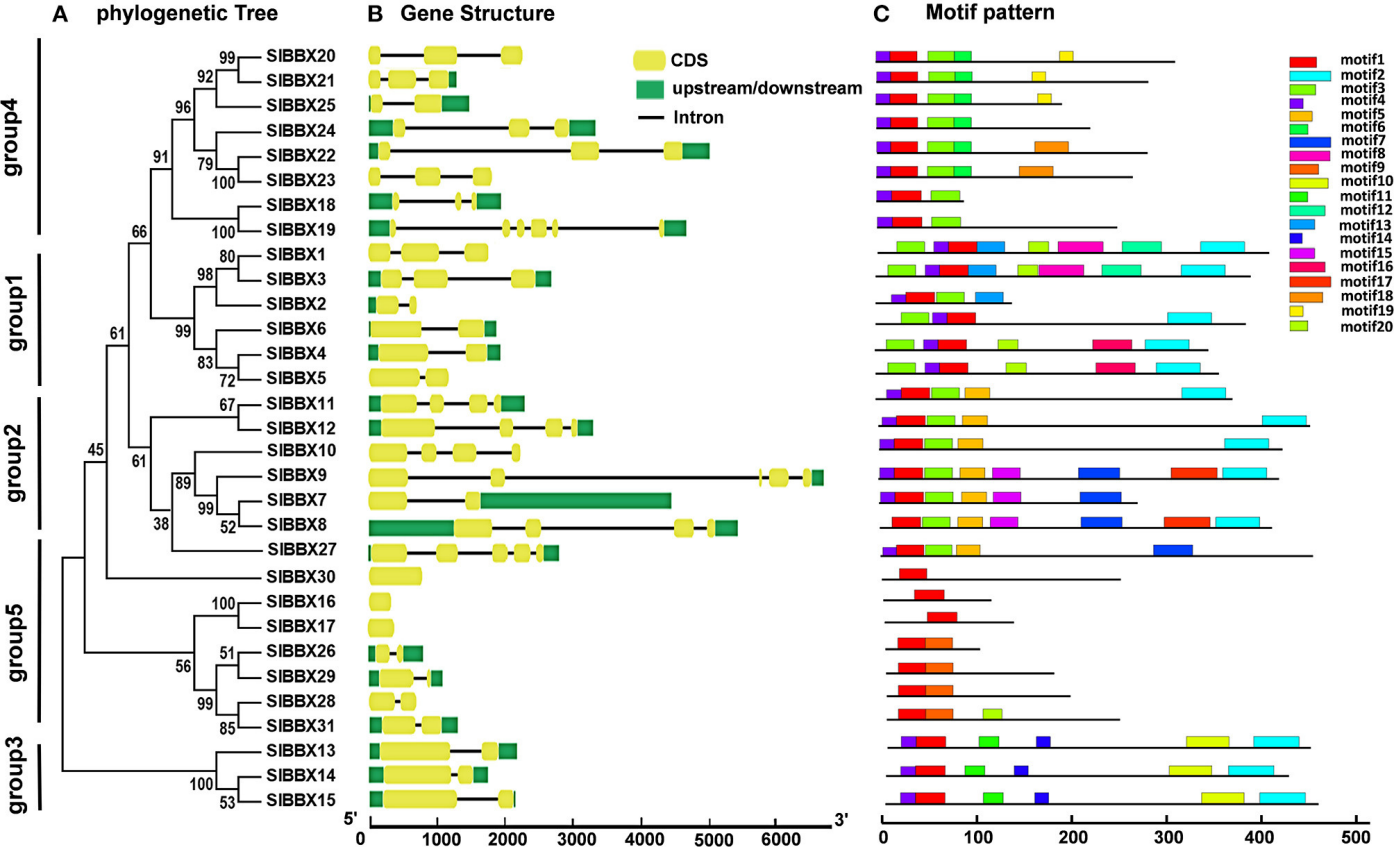
Phylogenetic relationship, gene structure and architecture of the conserved protein motifs in SlBBXs. **(A)** The phylogenetic tree was constructed based on the full-length sequences of SlBBX proteins. **(B)** Exon-intron structure of SlBBXs. Green boxes indicate untranslated 5′- and 3′-regions, yellow boxes indicate exons; and black lines indicate introns. **(C)** The motifs composition. The motifs, numbered 1–20, were displayed in different colored boxes. The sequence information for each motif is provided in Supplementary Table 1.

The evolution of multigene families can be driven by gene structural diversity. Examination of the genomic DNA sequences revealed that most *SlBBXs* contained one to five introns, while *SlBBX16, SlBBX17*, and *SlBBX30* had no introns (Figure 3B, Table 1, Supplementary Figure 3). Among them, nine *SlBBXs* had one intron, followed by 10 *SlBBXs* with two introns, five *SlBBXs* with three introns, three *SlBBXs* with four introns, and one *SlBBXs* with five introns. Generally, members of each subclass, which are most closely related, exhibited analogous exon-intron structures. For instance, the members in groups 1 and 4 had one to two, and zero to one intron, respectively (Figures 3A,B, Supplementary Figure 3). However, a few *SlBBX* genes showed dissimilar exon-intron arrangements. For instance, *SlBBX18* and *SlBBX19* had high sequence similarity, but *SlBBX18* and *SlBBX19* contained two and five introns, respectively (Figures 3A,B, Supplementary Figure 3). These divergences indicated that both the gain and loss of introns during evolution, may better explain the functional diversity of *SlBBX* homologous genes.

To further examine the structural features of SlBBXs, the conserved motif distributions were analyzed. Twenty conserved motifs were predicted (Figure 3C), while multilevel consensus sequences and the E-value of them were shown in Supplementary Table 1. The results showed that motif 17 was the largest motif depending on the width, followed by motif 8 and motif 2 (Supplementary Table 1). Motif 1 was found in all the SlBBXs (Figure 3C). Notably, 74.2% and 70.1% SlBBXs contained motif 4 and motif 3, respectively. Motif 2 was unique to the group 1, 2, and 3 of SlBBXs, while motif 5 was unique to group 2 except for the SlBBX27. Motif 10 was found only in group 3 of SlBBXs. Our results showed that members that are most closely related in the phylogenetic tree contained common motifs on the basis of alignment and position, which indicated that they may have a similar biological function.

**Figure 4:**
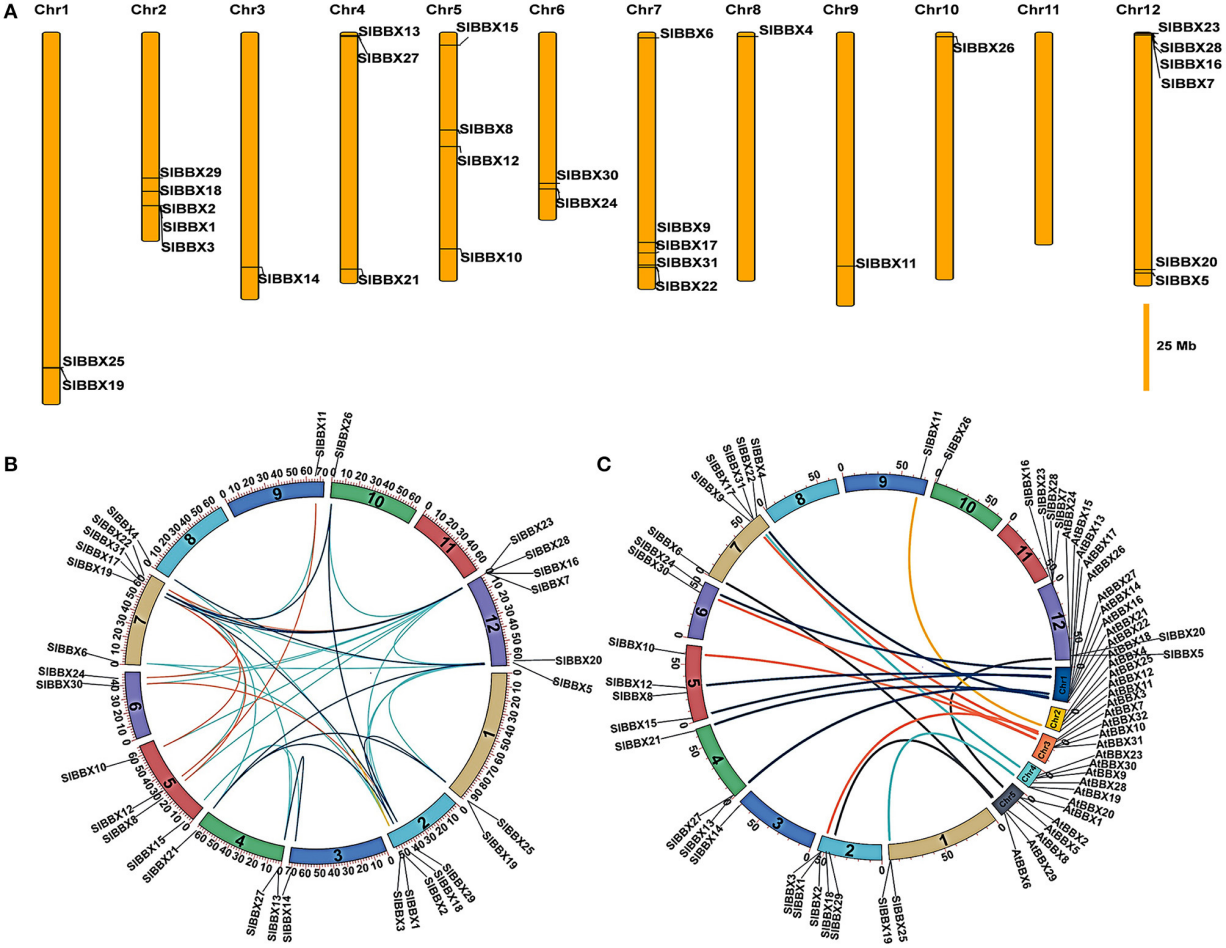
Chromosome distribution and syntenic analysis of *SlBBX* genes in tomato. **(A)** Positions of SlBBX genes family members on tomato chromosomes. **(B)** Segmental duplication of tomato *BBX* genes. Gene pairs located in the segmental duplicated chromosomal regions were linked using different lines. **(C)** Syntenic analysis of tomato and Arabidopsis *BBX* genes. Colored curves denote the syntenic relationships between tomato and Arabidopsis *BBX* genes.

Chromosomal locations showed that 31 *SlBBX* genes were unevenly distributed on the 12 chromosomes (Figure 4A). A maximum number of *SlBBX* genes were found on chromosome 12 (Chr12), comprising six genes. Five genes were located on Chr2 and Chr7. Four and three *SlBBX* genes were located on Chr5 and Chr4, respectively. Both Chr1 and Chr6 contained two members of *SlBBX* genes, whereas only one gene was detected on Chr3, 8, 9, and 10. Additionally, no *SlBBX* genes were found on Chr11.

Thirty-six pairs of *SlBBX*s were identified as segmental duplication in the tomato genome (Figure 4B). Chr2, 7, and 12 had more duplication regions, which partially explain the greater numbers of *SlBBX* genes that were located on these three chromosomes. Although *SlBBX1* and *SlBBX3* were located on the same chromosome (Figure 4A), and their sequence identity was 83% (Supplementary Figure 4), they were not tandem duplication. To further examine the evolutionary relationships between *SlBBX*s and *AtBBXs*, a synteny analysis was performed. A total of 16 of *SlBBX*-*AtBBX* orthologous pairs were identified (Figure 4C), which indicated the existence of numerous *SlBBX* genes prior to the divergence of Arabidopsis and tomato. Some members of *SlBBXs* were not localized in the syntenic block, suggesting that these genes might have certain specificity due to their evolution time.

### Analysis of *cis*-Elements in the Promoter Region of *SlBBXs*

**Figure 5:**
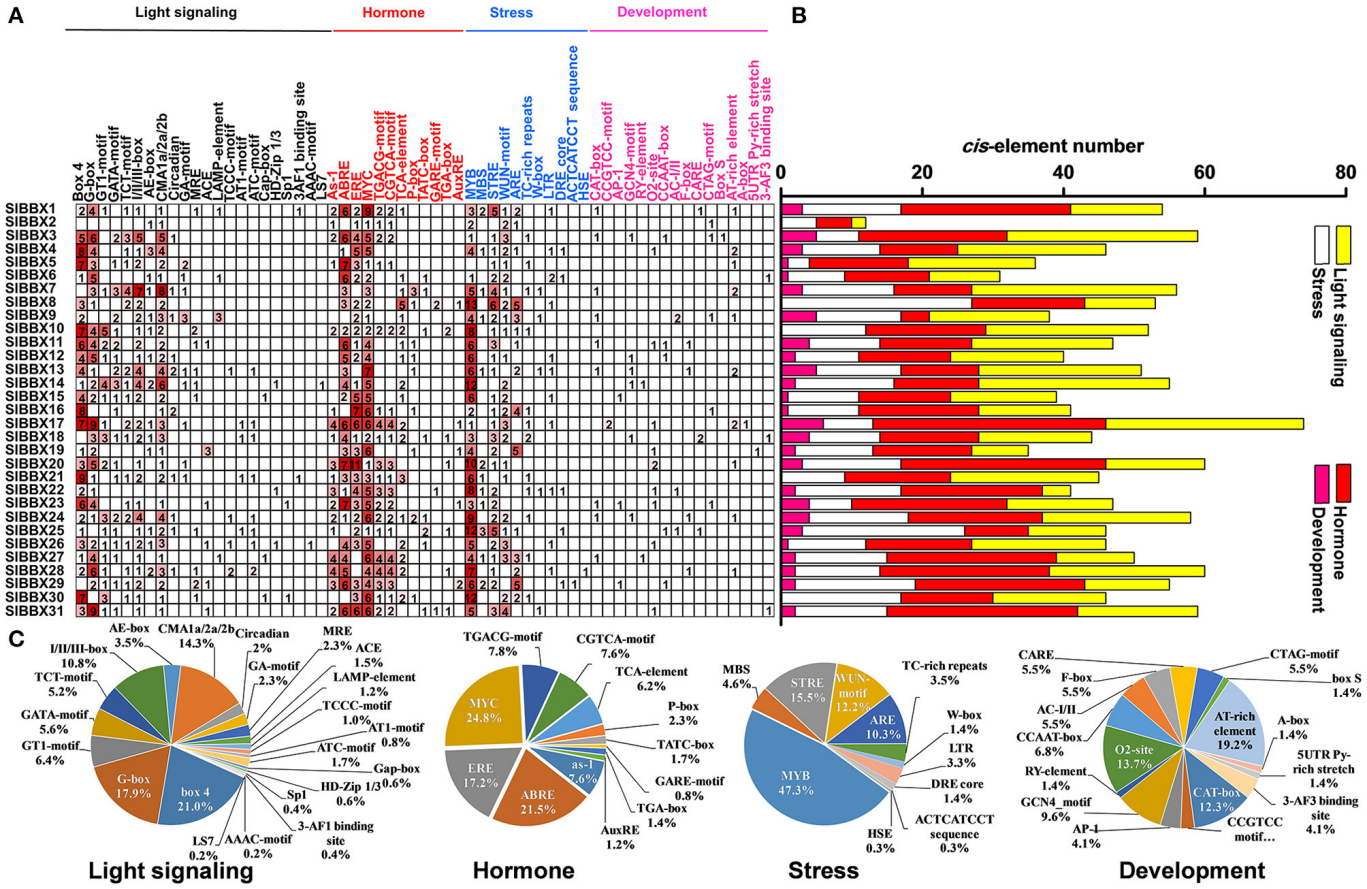
Inspection of *cis*-acting elements in tomato *BBX* genes. **(A)** The numbers of different promoter elements in these *SlBBX* genes were indicated by different colors and numbers of the grid. **(B)** The sum of the *cis*-acting elements in each category was represented by different colored histograms. **(C)** Pie charts with different sizes represented the ratio of each promoter element in each category.

A total of 61 major *cis*-elements were predicted in promters of *SlBBX* genes (Figure 5A), including 22 light responsive, 12 hormone responsive, 11 stress responsive, and 16 development. The number of light responsive *cis*-elements was the largest in the promoters of 31 *SlBBX* genes (Figure 5B). The number of *cis*-elements in the promoters of *SlBBX17* and *SlBBX2* was the largest and least, respectively. The major light responsive elements contained box4 (21%), G-box (17.9%), and CMA1a/2a/2b (14.3%), which were located on 87.1% (27/31), 83.9% (26/31), and 96.8% (30/31) of *SlBBX*s promoters, respectively (Figure 5C). The most common motifs were the JA-responsive elements (MYC), abscisic acid (ABA)-responsive element (ABRE), and ethylene-responsive element (ERE), accounting for 24.8%, 21.5%, and 17.2% of the scanned hormone responsive motifs, respectively. The stress responsive elements MYB, STRE (stress-related elements) and WUN were located on 96.8% (30/31), 90.3% (28/31), and 77.4% (24/31) of 31 *SlBBX* genes promoters, respectively. In the development category, various growth and development related elements, such as AT-rich element (19.2%), O_2_-site for zein metabolism regulation (13.7%), CAT-box for meristem expression (12.3%), GCN4_motif required for endosperm expression (9.6%), were found. Our findings suggest that the promoter regions of *SlBBX* genes that contained the *cis*-elements played a critical role in the light and stress responses.

### *SlBBX* Genes Expression in Response to Different Light Quality

**Figure 6:**
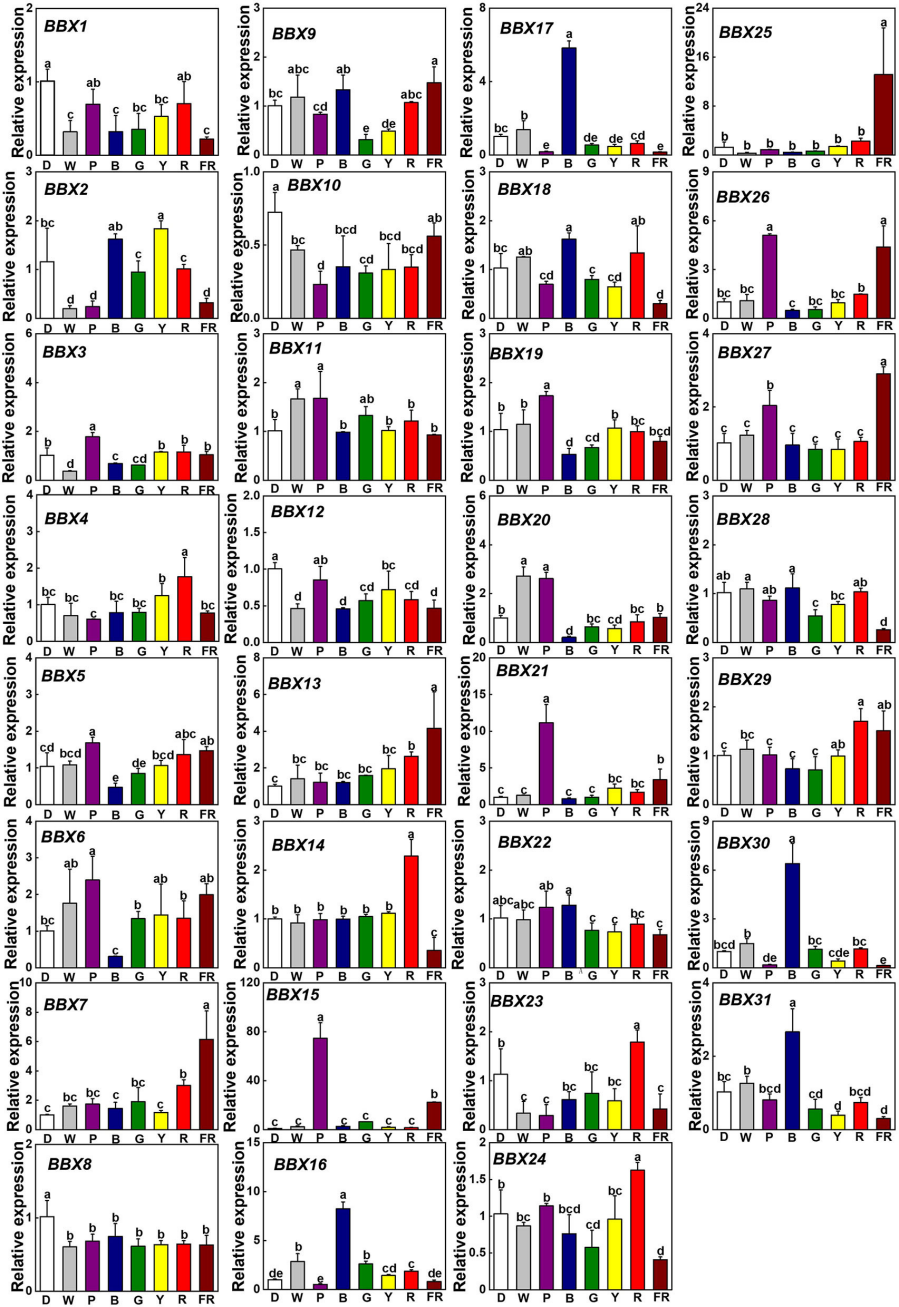
Gene expression of *SlBBXs* in tomato leaves after the exposure of plants to different light quality for 6 h from the dark. Light quality treatments include dark (D), white light (W) or purple (P), blue (B), green (G), yellow (Y), red (R), and far-red (FR) light. The light intensity was 100 μmol m^−2^ s^−1^. Data are presented as the means of three biological replicates (±SD). Different letters indicate significant differences (*P* < 0.05) according to Tukey's test.

To assess whether light signaling regulates *SlBBXs*, we investigated the gene expression of *SlBBXs* in tomato plants grown at dark (D), white (W), and different light quality [purple (P), blue (B), green (G), yellow (Y), red (R), and far-red (FR)] conditions. In comparison with D, light decreased the transcripts of *SlBBX1, SlBBX8, SlBBX10*, and *SlBBX12*, while it increased the transcripts of *SlBBX7, SlBBX13*, and *SlBBX15* (Figure 6). Plants grown at R light conditions showed higher expression of *SlBBX4, SlBBX14, SlBBX23, SlBBX24*, and *SlBBX29* than those grown at other light qualities. Whereas, FR light significantly up-regulated the transcripts of *SlBBX7, SlBBX13, SlBBX15, SlBBX21, SlBBX25, SlBBX26*, and *SlBBX27*, it obviously down-regulated the transcripts of *SlBBX14, SlBBX16, SlBBX18, SlBBX24, SlBBX28, SlBBX30*, and *SlBBX31* (Figure 6). Transcripts of *SlBBX16, SlBBX17, SlBBX18, SlBBX30*, and *SlBBX31* were induced, while transcripts of *SlBBX5, SlBBX6, SlBBX19*, and *SlBBX20* were inhibited in plants when grown at B light conditions. *SlBBX15* was induced by G light irradiation at 6 h, whereas *SlBBX9* and *SlBBX28* were repressed (Figure 6). Y light led to an obvious reduction in expression of *SlBBX9* and *SlBBX31*. Obviously, the P light increased the expression of *SlBBX3, SlBBX5, SlBBX6, SlBBX15, SlBBX19, SlBBX20, SlBBX21, SlBBX26*, and *SlBBX27*, but decreased the expression of *SlBBX10* and *SlBBX16*. Interestingly, *SlBBX4, SlBBX23*, and *SlBBX29* were only responsive to R light, while *SlBBX7, SlBBX13*, and *SlBBX25* were induced just in response to FR light. Meanwhile, R light induced the expression of *SlBBX14* and *SlBBX24*, but FR light inhibited their expression (Figure 6). In general, the results showed that *SlBBX* genes might act a critical role in response to light quality signaling.

### *SlBBXs* Act Critical Roles in Regulation of Cold Tolerance in Tomato

**Figure 7:**
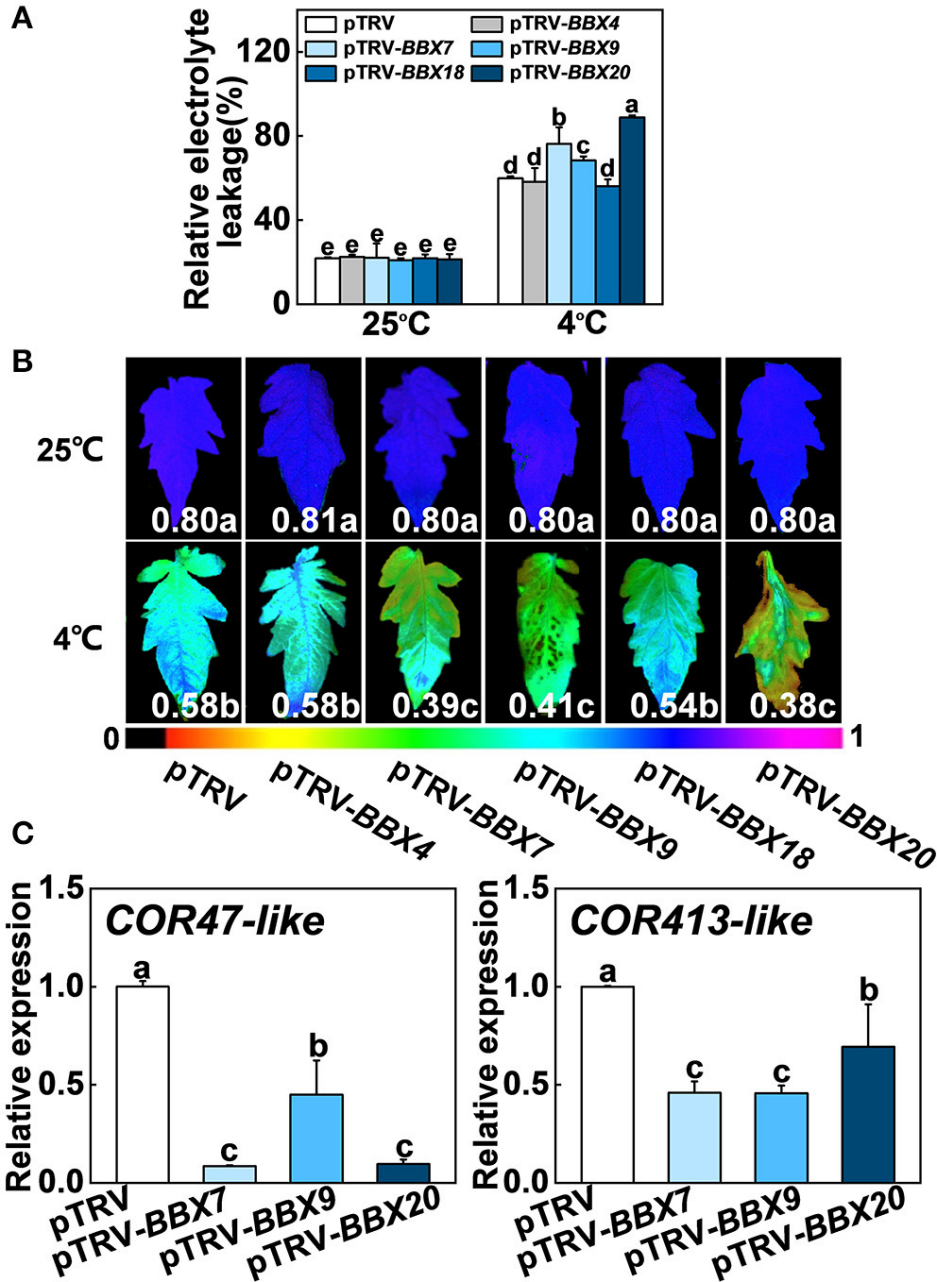
Roles of *SlBBXs* in cold tolerance in tomato plants. **(A,B)** The relative electrolyte leakage **(A)** and the maximum quantum yield of PSII (Fv/Fm) **(B)** in tomato wild-type (pTRV) and *BBXs*-silenced plants (pTRV-*BBXs*) after exposure to cold stress for 7 d. The false color code depicted at the bottom of the image ranges from 0 (black) to 1.0 (purple), representing the level of damage in leaves. **(C)** Cold responsive gene expression in tomato plants exposed 4°C for 6 h. Data are presented as the means of three biological replicates (±SD). Different letters indicate significant differences (*P* < 0.05) according to Tukey's test.

To investigate whether *SlBBX* genes participated in cold stress, we analyzed the relative expression data of *SlBBX* genes in tomato plants (Chu et al., 2016), chose five genes, including *SlBBX4, SlBBX7, SlBBX9, SlBBX18*, and *SlBBX20*, and performed virus-induced gene silencing (VIGS) experiments to study their function under cold stress. After cold stress, the levels of relative electrolyte leakage (REL) in *SlBBX7*-silenced plants (pTRV-*BBX7*), *SlBBX9*-silenced plants (pTRV-*BBX9*) and *SlBBX20*-silenced plants (pTRV-*BBX20*) were higher than wild-type (pTRV) (Figure 7A), meanwhile, these silenced plants showed an increased sensitivity to cold stress compared with pTRV, as evidenced by a decrease in the maximum photosystem II efficiency (Fv/Fm) (Figure 7B, Supplementary Figure 5). In addition, the transcription of *COLD RESPONSIVE (COR)* genes, including *COR47-like COR413-like* was markedly lower in *SlBBXs*-silenced plants than those in control plants (pTRV) under cold stress (Figure 7C), which indicated that *SlBBX7, SlBBX9*, and *SlBBX20* induced cold responsive genes under cold stress. Together, these results indicated that *SlBBX7, SlBBX9*, and *SlBBX20* play positive roles in cold tolerance in tomato plants.

### Roles of *SlBBXs* in Alleviation of Photoinhibition Under Cold Stress in Tomato

**Figure 8:**
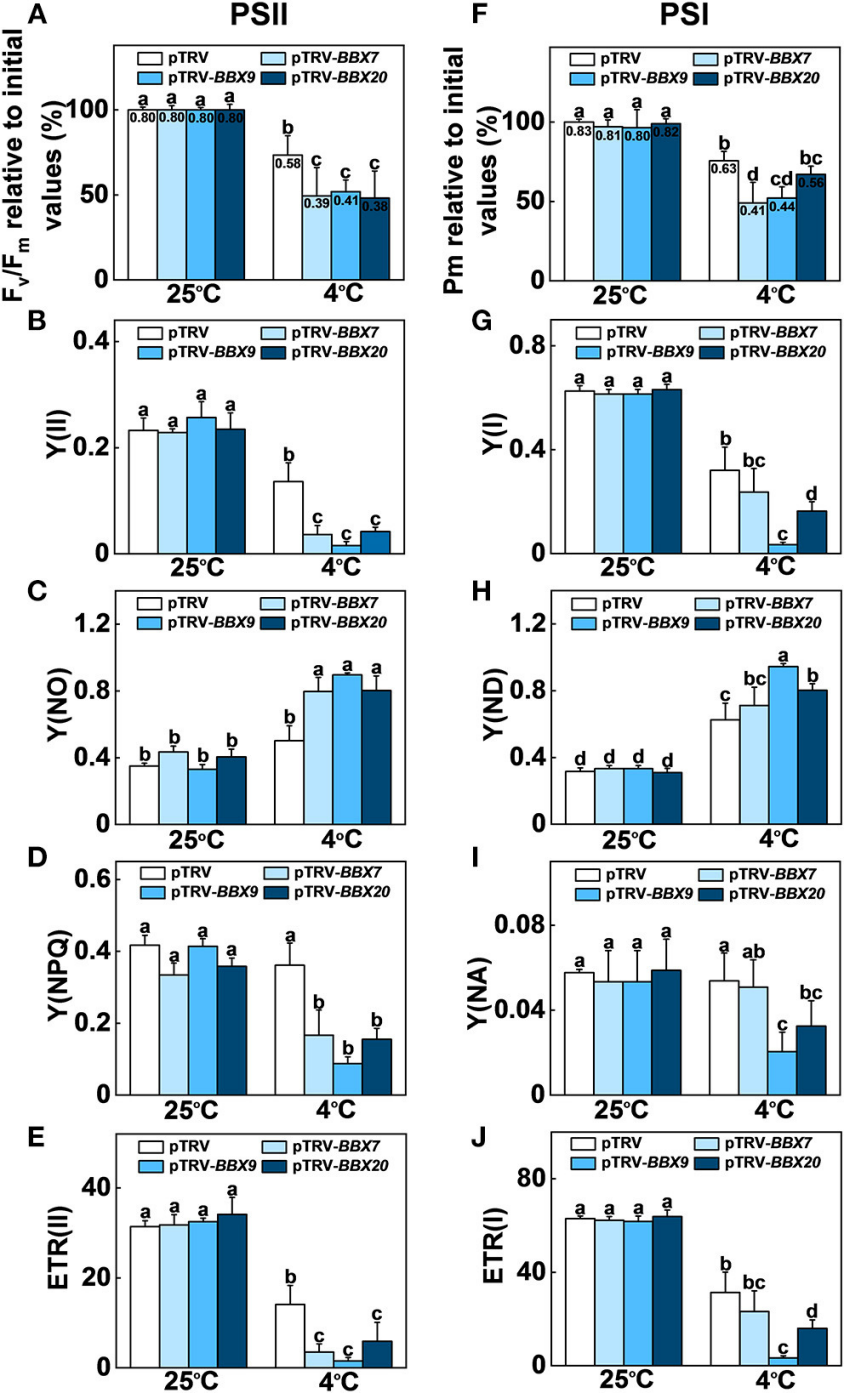
*SlBBXs* play important roles in alleviating cold-induced photoinhibition in tomato plants. **(A–E)** Changes in PSII parameters, including Fv/Fm **(A)**, Y(II) **(B)**, Y(NO) **(C)**, Y(NPQ) **(D)**, and ETR(II) **(E)** in tomato wild-type (pTRV) and *BBXs*-silenced plants (pTRV-*BBXs*) after exposure to cold stress for 5 d. **(F–J)** Changes in PSI parameters, including Pm **(F)**, Y(I) **(G)**, Y(ND) **(H)**, Y(NA) **(I)**, and ETR(I) **(J)** in tomato wild-type and *BBXs*-silenced plants after exposure to cold stress for 5 d. Data are presented as the means of three biological replicates (±SD). Different letters indicate significant differences (*P* < 0.05) according to Tukey's test.

We analyzed the roles of *SlBBXs* in cold-induced photoinhibition. The maximum quantum yield of PSII (Fv/Fm) and the maximum level of the P700 signal (Pm, full oxidation of P700) in the dark were measured before and after cold treatment. Before treatment, Fv/Fm and Pm were similar among *SlBBX7-, SlBBX9-, SlBBX20-* silenced plants, and WT (pTRV) plants (Figures 8A,F). Fv/Fm was decreased by 27% in the pTRV plants after cold stress, while it was decreased by 51%, 48%, and 52% in *SlBBX7-, SlBBX9-*, and *SlBBX20-* silenced plants, respectively, after cold stress, which indicated that disruption of these three *SlBBX*s caused photoinhibition of PSII during cold stress. Furthermore, the Pm was decreased by 25% after cold stress, whereas it was decreased by 51%, 48%, and 33%, respectively, in *SlBBX7-, SlBBX9-*, and *SlBBX20-* silenced plants after cold stress, which suggested that the disruption of *SlBBX7* and *SlBBX9* caused obvious photoinhibition of PSI after cold stress, but the photoinhibition of PSI was slight in *SlBBX20-* silenced plants after cold stress. Together, these results indicated that these three SlBBXs play important roles in alleviating the photoinhibition of both photosystems during cold stress.

**Figure 9:**
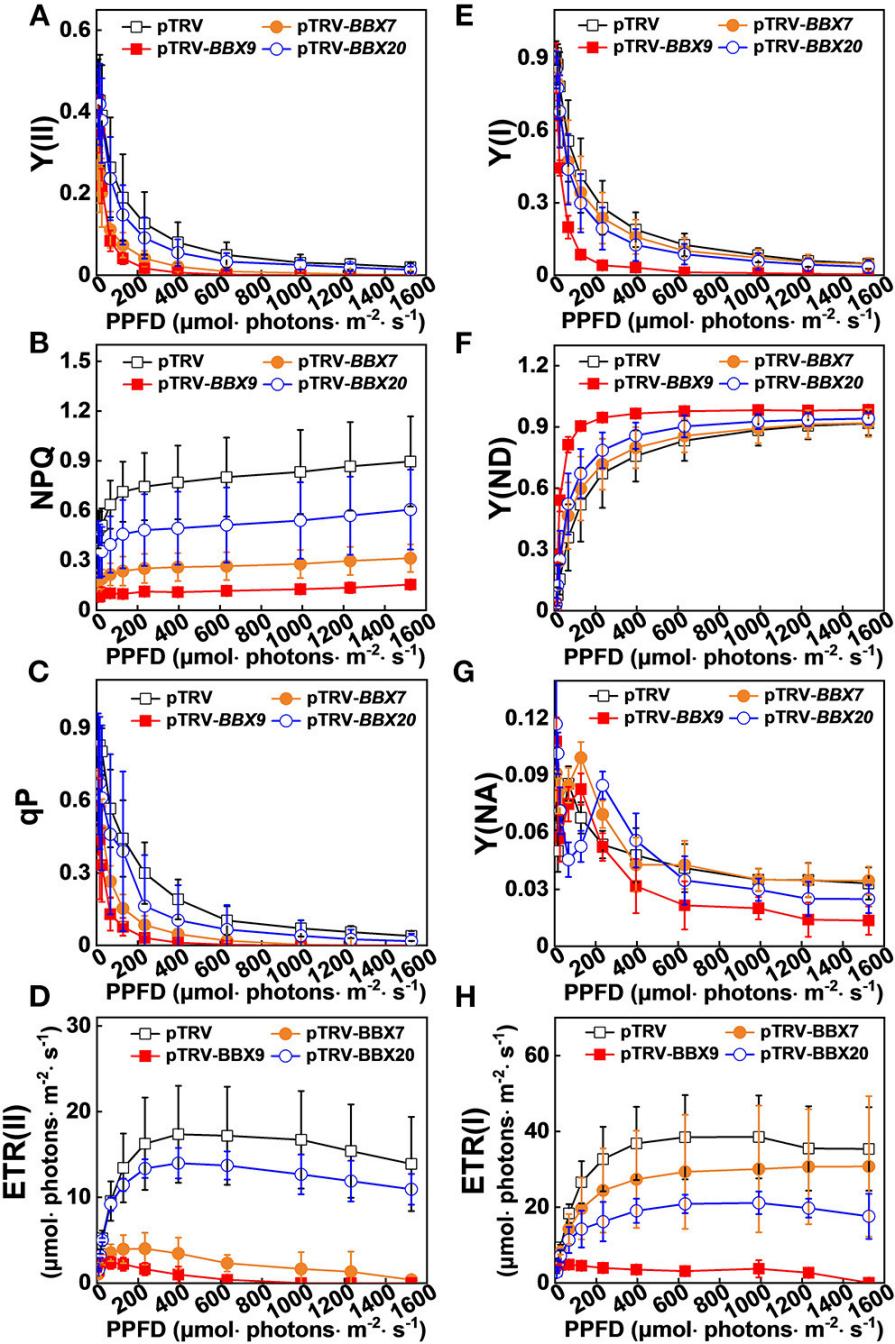
Impact of disruption of *SlBBXs* on the electron transport during steady-state photosynthesis in tomato plants grown under cold stress. The light intensity dependence of PSII and PSI photosynthetic parameters was monitored in the tomato wild type (WT) and *BBXs*-silenced plants (pTRV-*BBXs*). **(A–D)** PSII parameters Y(II) **(A)**, NPQ **(B)**, qP **(C)**, and ETR(II) **(D)** in tomato wild-type (pTRV) and *BBXs*-silenced plants (pTRV-*BBXs*) after exposure to cold stress for 5 d, respectively. **(E–H)** PSI parameters Y(I) **(E)**, Y(ND) **(F)**, Y(NA) **(G)**, and ETR(I) **(H)** in tomato wild-type (pTRV) and *BBXs*-silenced plants (pTRV-*BBXs*) after exposure to cold stress for 5 d, respectively. Data are presented as the means of three biological replicates (±SD). Different letters indicate significant differences (*P* < 0.05) according to Tukey's test.

In order to obtain a more detailed insight into the processes affecting photoinhibition in the *SlBBX*-silenced plants after cold stress, some electron transport parameters of photosystems I and II were measured. Both ETR II (Figures 8E, 9D) and ETR I (Figures 8J, 9H) were significantly reduced after cold stress in tomato plants, showing a decrease of 50%–55% over those in plants grown at normal temperature. The ETR II and ETR I in *SlBBXs*-silenced plants were lower than those in control plants (pTRV) after cold stress, except *SlBBX7*-silenced plants, whose ETR I is close to the values of pTRV. To further explore the decrease in the ETRs, we measured Y(II), Y(NPQ), and Y(NO) for PSII and Y(I), Y(ND), and Y(NA) for PSI. We found that Y(II) values were significantly lower in pTRV-*BBX7*, pTRV-*BBX9*, and pTRV-*BBX20* plants than those in pTRV plants under cold stress (Figures 8B, 9A). This decrease in Y(II) appeared to be primarily due to a decrease in quantum yield of regulated energy dissipation of PSII [Y(NPQ)] and photochemical quenching coefficient (qP), and an increase in quantum yield of non-regulated energy dissipation of PSII [Y(NO)] in *SlBBXs*-silenced plants, especially in pTRV-*BBX7* and pTRV-*BBX9* plants (Figures 8B–D, 9A–C). Like Y(II), Y(I) was also decreased in *SlBBXs-*silenced plants, except for in pTRV-*BBX7* plants (Figures 8G, 9E). These decrease seemed to be due to obvious donor side limitation of PSI (due to lower ETR II in PSII) as reflected by the elevated Y(ND), which were 29% and 47% higher in pTRV-*BBX9* and pTRV-*BBX20* plants, respectively, than in pTRV plants (Figures 8H, 9F). Although Y(ND) was 14% higher in pTRV-*BBX7* plants than in pTRV plants, its Y(I) was similar to the control plants (pTRV), which indicated that disruption of *SlBBX7* damaged the PSII rather than PSI during cold stress. Therefore, cold stress seriously reduced the capacity for photochemical energy conversion, electron transport rate and photoprotection in *SlBBX7-, SlBBX9-, SlBBX20-* silenced plants, suggesting that these three SlBBXs play critical roles in alleviating photoinhibition under cold stress.

## Discussion

In this study, we identified and characterized 31 *SlBBX* genes in tomato (Figure 1, Tables 1, 2), which contained two additional loci encoding BBX proteins in the tomato genome, that were named *SlBBX30* and *SlBBX31*, in comparison with the previous studies (Chu et al., 2016). BBX proteins are characterized by one or two B-box domains at the N-terminal and, in some cases, a CCT domain at the C-terminal (Gangappa and Botto, 2014). Here, we found both the newly retrieved SlBBX proteins (SlBBX30 and SlBBX31) contain a B-box domain at the N-terminal (Figure 1, Table 2), and they were also clustered in group 5 (Figure 2, Supplementary Figure 2), which further indicated these two proteins were new SlBBX proteins. The release of new tomato genomes and database updates may be the primary causes of this phenomenon. There were five subfamilies in the 32 members of Arabidopsis BBXs according to the combination of different conserved domains (Khanna et al., 2009). However, the conserved domain-based classification of BBX proteins in tomato was rather complex. As shown in Figure 2, SlBBX1 to SlBBX6 were classified into group 1, which had two B-boxes and a CCT plus a VP domains, whereas SlBBX1 lacked a VP domain, and SlBBX2 only contained two B-boxes (Table 2). Meanwhile, SlBBX7 to SlBBX12 and SlBBX27 were clustered into group 2, which possessed two B-boxes and a CCT domains; however, SlBBX8 and SlBBX10 had one B-box and a CCT domains, while SlBBX7 and SlBBX27 contained two B-boxes. Group 4 contained only one B-box. We investigated the detail of sequence alignment in SlBBXs (Figure 1), and found a high degree of conservation of the B-box1 domain among SlBBX7 to SlBBX12, thus the clustering results of these proteins were similar to that based on B-box1. These results indicated that during the process of evolution, some SlBBX proteins lost the B-box2 domain.

Accumulating evidence showed that some BBX proteins act as central players in a variety of light-regulated physiological processes in plants. Here, we found that the number of light responsive *cis*-elements was the largest in the promoters of 31 *SlBBX* genes (Figure 5), which indicated that *SlBBX* genes were regulated by light signaling. Thus, we examined the gene expression of all the *SlBBXs* in response to different light quality. Results showed that light decreased the transcripts of *SlBBX1, SlBBX8, SlBBX10*, and *SlBBX12*, while increased the transcripts of *SlBBX7, SlBBX13*, and *SlBBX15* compared with dark (Figure 6). Previous studies had demonstrated that COP1, which is degraded after illumination, works as an E3 ubiquitin ligase that targets a variety of light signaling factors for ubiquitination and degradation in darkness (Osterlund et al., 2000; Han et al., 2020). For example, COP1 interacts with multiple BBXs, such as CO/BBX1 and BBX10, and subsequently degrades them by the 26S proteasome system (Liu et al., 2008; Ordoñez-Herrera et al., 2018). Nevertheless, COP1 stabilizes BBX11 rather than degrading it (Zhao et al., 2020), which suggests that COP1 likely degrades a yet unidentified component(s) targeting BBX11. Thus, COP1 may also control the stability of SlBBX proteins, including SlBBX1, SlBBX7, SlBBX8, SlBBX10, SlBBX12, SlBBX13, and SlBBX15, in the transition from dark to light. Interestingly, we found that *SlBBX4, SlBBX23*, and *SlBBX29* were expressed only in response to R light, while *SlBBX7, SlBBX13*, and *SlBBX25* were expressed just in response to FR light (Figure 6). These results indicate that these SlBBX proteins might directly interact with the photoreceptors, which sense R and FR light signals. Similarly, recent work has revealed that phyB directly interacts with BBX4 and positively regulates its accumulation in red light in Arabidopsis (Heng et al., 2019a), which demonstrates that photoreceptors may directly control some BBX proteins. In addition, the results showed that R light induced the expression of *SlBBX14* and *SlBBX24*, whereas FR light inhibited their expression (Figure 6), which implied that these two SlBBX proteins might function antagonistically to regulate some plant physiological processes, such as shade avoidance and the elongation of hypocotyls. Here, we observed that B light induced the gene expression of *SlBBX16, SlBBX17, SlBBX18, SlBBX30*, and *SlBBX31*, whereas inhibited the transcripts of *SlBBX5, SlBBX6, SlBBX19*, and *SlBBX20* (Figure 6). Previous work demonstrated that *BBX28*/*BBX29* and BBX30/BBX31 could precisely control each other by forming a feedback loop in Arabidopsis (Lin et al., 2018; Heng et al., 2019b; Yadav et al., 2019; Song et al., 2020). Thus, these SlBBX proteins may work in concert with each other and some unidentified factors to regulate the plant growth in response to light signaling.

Light and temperature are more or less inter-related during plant growth and stress response (Wang et al., 2016). Here, we observed that the disruption of *SlBBX7, SlBBX9*, and *SlBBX20* largely reduced the cold tolerance in tomato plants as evidenced by phenotypes, REL, Fv/Fm, and cold responsive genes (Figure 7, Supplementary Figure 5), which indicated that *SlBBX7, SlBBX9*, and *SlBBX20* positively regulate cold tolerance in tomato plants. In addition, far-red light (FR) induced the transcription of *SlBBX7* and *SlBBX9* (Figure 6). and enhanced the cold tolerance in tomato plants (Wang et al., 2016, 2018, 2019, 2020a,b), which indicate that SlBBXs may play critical roles in the links between cold response and light signaling. Recent studies also showed that BBX18 and BBX23 are involved in the thermomorphogenesis in Arabidopsis (Ding et al., 2018). Both *MdBBX20* and *MaCOL1* are responsive to low temperature in apple and banana, respectively (Chen et al., 2012; Fang et al., 2019). *ZFPL*, a homologous gene of *AtBBX32*, enhances cold tolerance in the grapevine (Takuhara et al., 2011). *CmBBX24* also increases plant cold tolerance in *Chrysanthemum* (Yang et al., 2014). Furthermore, MdBBX37 positively regulates JA-mediated cold-stress resistance in apples (An et al., 2021). However, whether BBXs are involved in cold stress–induced photoinhibition and the regulation of photoprotection during cold stress remains elusive.

Interestingly, the impairment of *SlBBX7, SlBBX9*, and *SlBBX20* significantly suppressed the photochemical efficiencies and energy conversion in tomato plants under cold stress, leading to the overreduction of electron carriers and damage of photosystem in tomato plants (Figures 8, 9). More recently, it has been demonstrated that heterologous expression of Arabidopsis *BBX21* in potato plants increases photosynthetic efficiency and reduces photoinhibition (Crocco et al., 2018). Therefore, BBXs play critical roles in photosynthesis and photoinhibition, however, the regulation mechanism is poorly understood. Previous works have revealed that BBX20, BBX21, BBX22, and BBX23 interact with HY5 to increase its transcriptional activity toward the target genes (Datta et al., 2008; Zhang et al., 2017; Job et al., 2018), whereas BBX24, BBX25, BBX28, and BBX29 suppress HY5 activity in Arabidopsis (Gangappa et al., 2013; Job et al., 2018; Lin et al., 2018; Song et al., 2020). HY5 also positively controls *BBX22* at the transcriptional level (Chang et al., 2008), while repressing *BBX30* and *BBX31* gene expression by binding to the promoters of these two genes (Heng et al., 2019b; Yadav et al., 2019). In addition, direct interactions between BBX32 and BBX21 lead to inhibition in the BBX21-HY5 (Holtan et al., 2011). Therefore, SlBBXs might alleviate the photoinhibition in tomato plants during cold stress through an HY5-dependent photoprotection pathway. Our previous results demonstrated that SlHY5 alleviated photoinhibition in tomato plants under cold stress by induction of photoprotection, including increased NPQ, cyclic electron flux (CEF) around PSI and the activities of Foryer-Halliwell-Asada cycle enzymes (Wang et al., 2018). Here, we showed that the over-reduction in the flow of electrons from PSII to PSI and considerably low levels of NPQ caused a high excitation pressure in *SlBBXs*-silenced plants against cold stress, leading to a severe photoinhibition (Figures 8, 9). Thus, NPQ might function as a protective measure to prevent damage from high excitation pressure against photosynthesis and plant development (Upadhyay et al., 2013).

## Conclusions

In this study, SlBBX family genes were identified and characterized in tomato by systematic analysis of conserved domains, phylogenetic relationship, gene structure, chromosome location. Two new members, *SlBBX30* and *SlBBX31*, were identified from the newly released tomato genome sequences. The promoter responsive *cis*-acting regulatory elements and gene expression analysis indicated that multiple *SlBBX* genes were highly responsive to light quality and low temperature. Furthermore, we found that *SlBBX7, SlBBX9*, and *SlBBX20* positively regulate cold tolerance in tomato plants via the prevention of photoinhibition and enhancing photoprotection. Our study emphasized the positive roles of light signaling transcription factors SlBBXs in cold tolerance in tomato plants, which may improve the current understanding of the integration of light and temperature signals by plants to adapt to adverse environments.

## Data Availability Statement

The original contributions presented in the study are included in the article/Supplementary Material, further inquiries can be directed to the corresponding author/s.

## Author Contributions

FW and TL designed the research. FW, XB, XW, JY, YZ, SZ, and XS performed the experiments. FW, YL, MQ, and GA analyzed the data. FW, GA, and TL wrote and revised the paper. All authors have read and approved the manuscript.

## Conflict of Interest

The authors declare that the research was conducted in the absence of any commercial or financial relationships that could be construed as a potential conflict of interest.
