# Supplementary material for: Genome-Wide Characterization of B-Box Gene Family and Its Roles in Responses to Light Quality and Cold Stress in Tomato
Source: Front Plant Sci. 2021 Jul 5;12:698525. doi: 10.3389/fpls.2021.698525 (PMC8287887; doi:10.3389/fpls.2021.698525)
Supplement: Supplementary file 1 [file Data_Sheet_1.zip › Supplementary Figures.DOCX]

**FIGURE S1∣**The isoelectric point of *SlBBXs* containing different domains.


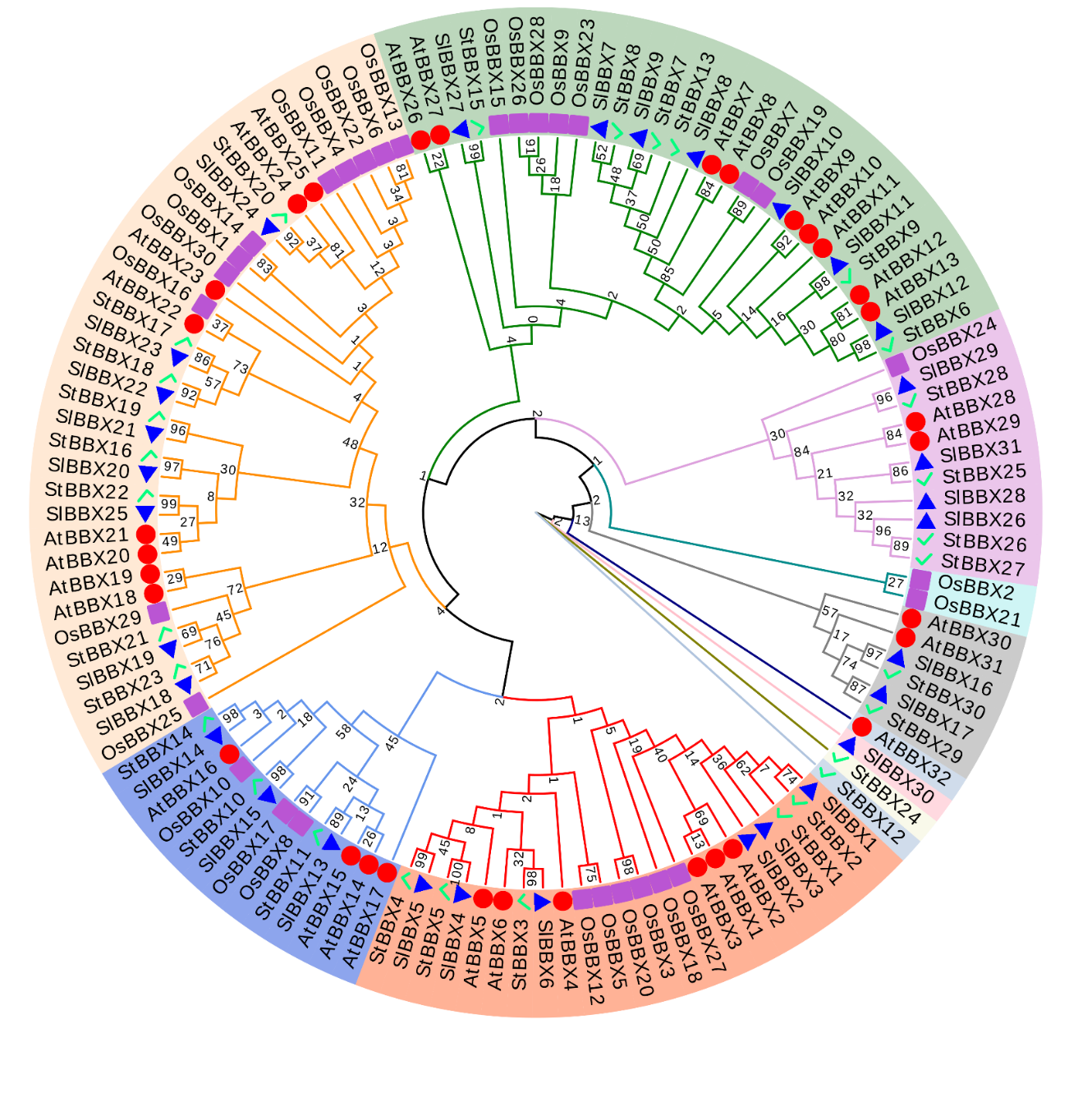


**FIGURE S2∣**Evolutionary relationships of tomato BBX family with Arabidopsis, rice and potato. The evolutionary history was inferred by using the Maximum Likelihood method based on the Jones-Taylor-Thornton (JTT) matrix-based model. The bootstrap consensus tree inferred from 1000 replicates is taken to represent the evolutionary history of the taxa analyzed. Branches corresponding to partitions reproduced in less than 50% bootstrap replicates are collapsed. Initial tree(s) for the heuristic search were obtained automatically by applying Neighbor-Join and BioNJ algorithms to a matrix of pairwise distances estimated using a JTT model, and then selecting the topology with superior log likelihood value. The analysis involved 123 amino acid sequences. All positions containing gaps and missing data were eliminated. There were a total of 30 positions in the final dataset. Evolutionary analyses were conducted in MEGA7. Gene family from each species has colored labels. Red circles, blue triangles, purple boxes, and green checkmark represent Arabidopsis, tomato, rice and potato, respectively.

**FIGURE S3∣**The intron characteristics of *SlBBXs* containing different domains.

**FIGURE S4∣**Multiple sequence alignment of the conserved domains of tomato SlBBX1 and SlBBX3. Completely conserved residues in a domain are indicated by black blue boxes.

**FIGURE S5∣**Phenotypes of *BBXs*-silenced (pTRV-*BBXs*) and non-silenced (pTRV) plants in tomato plants after exposure to 25 °C and 4 °C for 7 d.
