# Supplementary material for: Genome-Wide Characterization of B-Box Gene Family and Its Roles in Responses to Light Quality and Cold Stress in Tomato
Source: Front Plant Sci. 2021 Jul 5;12:698525. doi: 10.3389/fpls.2021.698525 (PMC8287887; doi:10.3389/fpls.2021.698525)
Supplement: Supplementary file 1 [file Data_Sheet_1.zip › Supplementary Tables.DOCX]

**TABLE S1∣**The sequence analysis and weblog of 20 identified motifs of the *SlBBX* genes family in tomato.

| Motifs | E-value | Sites | Width | Amino acid sequence | Logo |
| --- | --- | --- | --- | --- | --- |
| 1 | 3.1e-499 | 31 | 30 | YCRADEAALCWSCDRKVHSANKLASRHSRV | 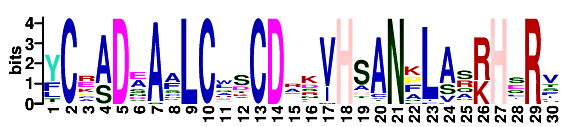 |
| 2 | 4.7e-340 | 13 | 46 | DREARVLRYREKKKTRKFEKTIRYASRKAYAETRPRIKGRFVKRTD | 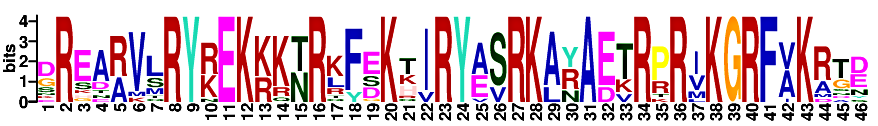 |
| 3 | 3.0e-201 | 21 | 29 | CDICQSSPAFVFCVEDRASLCRNCDWSIH | 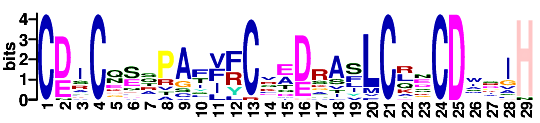 |
| 4 | 1.6e-69 | 23 | 15 | MKIVCDVCESARASV | 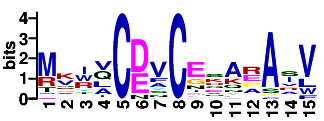 |
| 5 | 1.7e-35 | 7 | 26 | HKRQALSSYTGCPSASELSTIWSFLL | 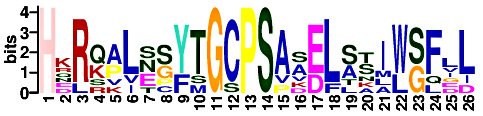 |
| 6 | 1.7e-23 | 6 | 19 | KANEHTQKHQRFLLTGVKV | 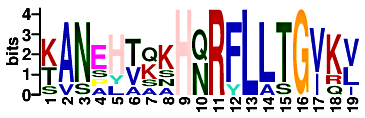 |
| 7 | 3.6e-23 | 4 | 43 | DNFNMDEVDLSIENYEELFGVSLDNPNQLFENEDIDSFFGMKD | 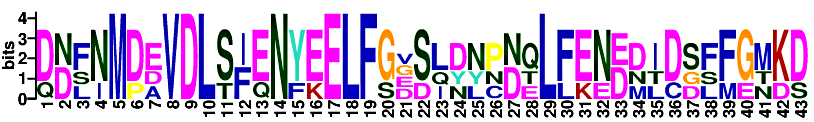 |
| 8 | 2.7e-20 | 2 | 47 | NNYGMLFGGEVVDDYLDLAEYGGDSQFNDQYSVNQQQQQYSVPQMSY | 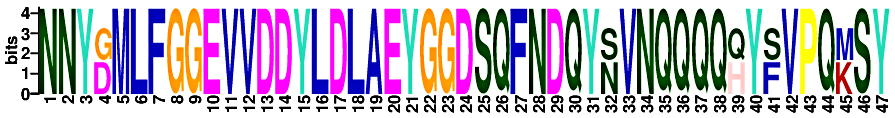 |
| 9 | 1.2e-18 | 4 | 30 | LLCNVCQSPTPWSASGAKLGPTVSVCQKCV | 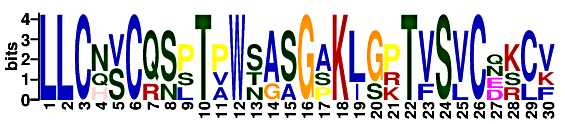 |
| 10 | 4.5e-17 | 3 | 43 | NKNKIMLNLDYESVLKAWPDQRSPWTNGVRPEVDSNDYWPDCM | 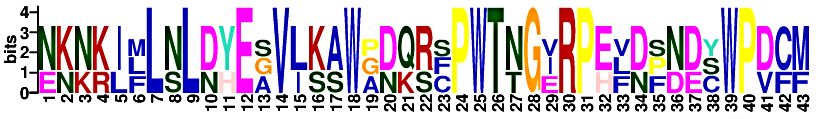 |
| 11 | 1.5e-16 | 3 | 20 | PSWHRGFTRKARTPRYGRKA | 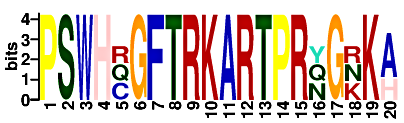 |
| 12 | 2.2e-12 | 2 | 41 | QTQQQQQSHHQNFQLGMEYDNSNTRYGYPASMSHSVSVVSM | 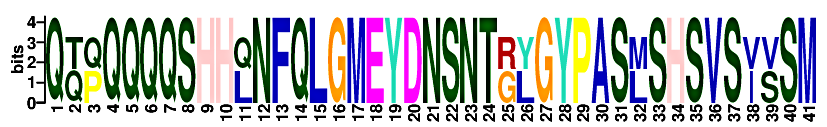 |
| 13 | 2.6e-21 | 3 | 29 | PIMTIPGTLYGPPAVETIGGGSMMIGGTT | 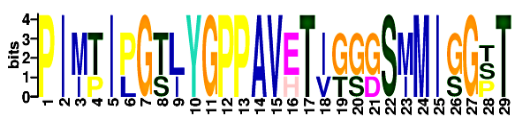 |
| 14 | 1.5e-10 | 3 | 14 | EEQLLYRVPIFDPF | 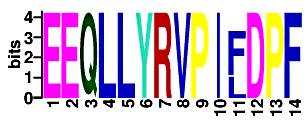 |
| 15 | 2.8e-14 | 3 | 29 | TCEQGMGSMSITDNRPTDSQHPQGKFNSQ | 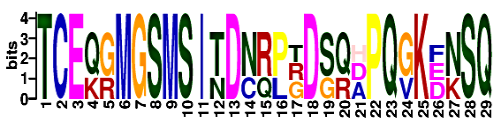 |
| 16 | 2.0e-10 | 2 | 41 | KPYMYNFTSQSISQSVSSSSMEVGVVPDHSTMTDVSNTFVR | 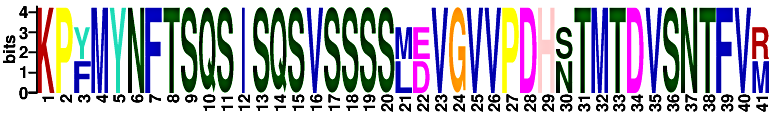 |
| 17 | 2.9e-09 | 2 | 48 | PTVCFARQQSSLSFSNLTGESNGGDYQECGASTMLRMGEPSWHPPCPE | 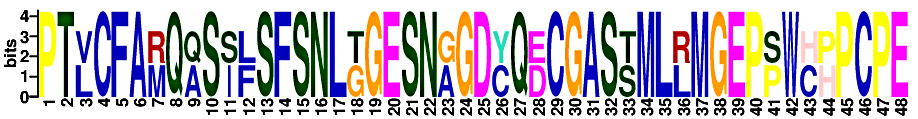 |
| 18 | 4.4e-09 | 2 | 37 | GSMPQWQFDQYLGMSDFNQNYGYMDYGSSKAGNGKVG | 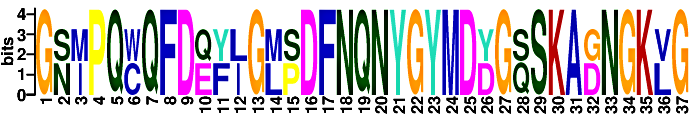 |
| 19 | 2.9e-08 | 3 | 15 | EYLEMLPGWHVEDFL | 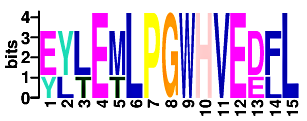 |
| 20 | 2.7e-07 | 5 | 21 | EDEDEAASWLLQNPPVKNNTK | 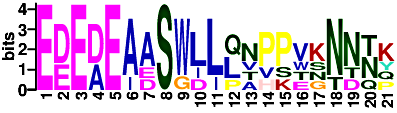 |

**TABLE S2∣**Primer sequences used for qRT-PCR analysis.

| **Gene** | **Accession number** | **Forward primer (5’-3’)** | **Reverse primer (5’-3’)** |
| --- | --- | --- | --- |
| ***SlBBX1*** | Solyc02g089520 | GTGACAGTTGCCGTTCAGTT | CGTCACAGTCTGCACACAAA |
| ***SlBBX2*** | Solyc02g089500 | CAACTGGTCAAGGGTGTGTG | CAGGCCTCACAAATCCACAC |
| ***SlBBX3*** | Solyc02g089540 | GTTTACTGTCGGGCGGATTC | CGAGAAGCCATGAGACTTGC |
| ***SlBBX4*** | Solyc08g006530 | ACGGAAGAAGAAGAAGCGGA | TACGGATCCACATCGCTGAA |
| ***SlBBX5*** | Solyc12g096500 | CAACCATTACAGCTCCGACG | AACGACTGGACCGGGTAAAT |
| ***SlBBX6*** | Solyc07g006630 | GTACCACAACCACAGCCACAAC | TGAAATCCATCCCTTTCATTTCTG |
| ***SlBBX7*** | Solyc12g006240 | CGAAGGGCTGCTATATGGGA | TCAAGTCCACCGCATCCATA |
| ***SlBBX8*** | Solyc05g020020 | ATGCTGTCGAGGGTTCATCA | GAGGATGGATGCTTGCCTTG |
| ***SlBBX9*** | Solyc07g045180 | AGTATTGTGGGGAGCAACGA | GACGTTGTGACAGGGCATTT |
| ***SlBBX10*** | Solyc05g046040 | AGGCTTTGCTTGCAATGTGA | TTCTTGAGTCGATGCCCTGT |
| ***SlBBX11*** | Solyc09g074560 | AACACACTCGTTGGTTGCTC | CCCCTCGAGTGGTCTTCTTT |
| ***SlBBX12*** | Solyc05g024010 | GCAGTTGAGGAGCCATGAAC | CATCATGGACAACGGAGCAG |
| ***SlBBX13*** | Solyc04g007210 | TGTGCATGGGAAATTGTGGG | ACCTTGTTCGTCGCTTTTCC |
| ***SlBBX14*** | Solyc03g119540 | TGGAATCCGACCCCATTTCA | ACCCTTGCTTCTCTTCCTCC |
| ***SlBBX15*** | Solyc05g009310 | CTTCTCCAACTACTCACAATCATC | GTTCGTCACTACTTCATCGTCTCC |
| ***SlBBX16*** | Solyc12g005750 | CAGAAGCCTATGTATATTGTGAAGCAG | TCATGTGAAACTCCAATCAAATACC |
| ***SlBBX17*** | Solyc07g052620 | GAGCTGATTTTGCCGACGAT | CGTTCTGTAGTCCGTCGTCT |
| ***SlBBX18*** | Solyc02g084420 | CCGTGCCTGTGATGAAAAGG | CGTTGGGCTTAGCAATTCCA |
| ***SlBBX19*** | Solyc01g110370 | CTGCCGTTCTTGTGACGAAA | ATGTCGCAACGCTGGATTTT |
| ***SlBBX20*** | Solyc12g089240 | GGATGAGGCTGCTCTTTGTG | AACCCCTCCTTTCCTGACAG |
| ***SlBBX21*** | Solyc04g081020 | GCAGCTTCATGTAGTGCGAA | ACAGAACCAGAAACAGGGGA |
| ***SlBBX22*** | Solyc07g062160 | CTCTGGACTTTACTGGCCCA | GGCTCGATGAAGGGGAGTAA |
| ***SlBBX23*** | Solyc12g005420 | CCTCCATCTGAAGGTGTATTAC | TTGCCTGCCTTCGATTGTCC |
| ***SlBBX24*** | Solyc06g073180 | TTAGCCACTGGAATCCGTGT | TTGTGGCTGCAGTTGGTTTT |
| ***SlBBX25*** | Solyc01g110180 | CTGGATCTGCTGCAAGTAATGCTGG | TGATGGAGAGTTGATCTGTGGAAC |
| ***SlBBX26*** | Solyc10g006750 | TAACCTTTGCTGGGATTGCG | GAAAGCTTTGGACCAGAGGC |
| ***SlBBX27*** | Solyc04g007470 | GTGAGCACATTCCCACGATC | GAAACCTCCTGATCGTTGGC |
| ***SlBBX28*** | Solyc12g005660 | TGACGAAGCAGCTGAGTTTG | TCTTCCGATAAGCTCCACCG |
| ***SlBBX29*** | Solyc02g079430 | GATGCCAGAGTTCACACAGC | CAGAAACAGTGGGACCAAGC |
| ***SlBBX30*** | Solyc06g063280 | GTCGACTTGTGTTTCCAGCA | TTCACCGTTAGAGTCGCTGT |
| ***SlBBX31*** | Solyc07g053140 | GTCAAATACGACGCCTCCAC | GAACTTCCGCTGTCGTCATC |
| ***COR47-like*** | Solyc04g082200 | TCTAGTAGCTCCAGTGATG | TCTCCTCTGTTTCCTCGT |
| ***COR413-like*** | Solyc02g071210 | AGCAGCTTATCTGGGTTT | AATGATGGCTAGGGTGGC |
| ***ACTIN2*** | Solyc11g005330 | TGTCCCTATTTACGAGGGTTATGC | CAGTTAAATCACGACCAGCAAGAT |

**TABLE S3∣**PCR primer sequences used for vector construction.

| **Vector** | **primer** | |
| --- | --- | --- |
| **pTRV2-*BBX4*** | **Forward** | 5′-CCGgaattcACAACTTCACCTCTCAATC-3′ |
|  | **Reverse** | 5′-CGCggatccTACGGAGAACGAAGTGGG-3′ |
| **pTRV2-*BBX7*** | **Forward** | 5′-CCGgaattcCGTCTATGCCTACTTTTG-3′ |
|  | **Reverse** | 5′-TCCcccgggGCACCTTTTATGTCCTTC-3′ |
| **pTRV2-*BBX9*** | **Forward** | 5′-CCGgaattcGGGTCAGCAGTGGGAAAG-3′ |
|  | **Reverse** | 5′-CGCggatccGCAAAGCAAAGGGTAGGAG-3′ |
| **pTRV2-*BBX18*** | **Forward** | 5′-CCGgaattcCACAGGTTAAGAAGGGAA-3′ |
|  | **Reverse** | 5′-CGCggatccGCACAGAAAAGAATGGCT-3′ |
| **pTRV2-*BBX20*** | **Forward** | 5′-CCGgaattcATTGGTGATGAGGATATGT-3′ |
|  | **Reverse** | 5′-CGCggatccCTGAGTCTCTTGCTTTGA-3′ |

The restriction enzyme cutting sites were indicated in red.
